# Factor quinolinone inhibitors alter cell morphology and motility by destabilizing interphase microtubules

**DOI:** 10.1038/s41598-021-02962-0

**Published:** 2021-12-07

**Authors:** Patrick Stoiber, Pietro Scribani Rossi, Niranjana Pokharel, Jean-Luc Germany, Emily A. York, Scott E. Schaus, Ulla Hansen

**Affiliations:** 1 MCBB Graduate Program, Boston University, Boston, MA 02215 USA; 2 Department of Biology, Boston University, Boston, MA 02215 USA; 3 Department of Chemistry, Boston University, Boston, MA 02215 USA; 4 Center for Molecular Discovery, Boston University, Boston, MA 02215 USA; 5 Present Address: Faculty of Medicine and Dentistry, Sapienza University of Rome, 00185 Rome, Italy

**Keywords:** Drug development, Mechanism of action

## Abstract

Factor quinolinone inhibitors are promising anti-cancer compounds, initially characterized as specific inhibitors of the oncogenic transcription factor LSF (*TFCP2*). These compounds exert anti-proliferative activity at least in part by disrupting mitotic spindles. Herein, we report additional interphase consequences of the initial lead compound, FQI1, in two telomerase immortalized cell lines. Within minutes of FQI1 addition, the microtubule network is disrupted, resulting in a substantial, although not complete, depletion of microtubules as evidenced both by microtubule sedimentation assays and microscopy. Surprisingly, this microtubule breakdown is quickly followed by an increase in tubulin acetylation in the remaining microtubules. The sudden breakdown and partial depolymerization of the microtubule network precedes FQI1-induced morphological changes. These involve rapid reduction of cell spreading of interphase fetal hepatocytes and increase in circularity of retinal pigment epithelial cells. Microtubule depolymerization gives rise to FH-B cell compaction, as pretreatment with taxol prevents this morphological change. Finally, FQI1 decreases the rate and range of locomotion of interphase cells, supporting an impact of FQI1-induced microtubule breakdown on cell motility. Taken together, our results show that FQI1 interferes with microtubule-associated functions in interphase, specifically cell morphology and motility.

## Introduction

Cell migration is a fundamental biological process at the heart of orchestrating the organization and rearrangement of cells and tissues into complex multi-cellular structures. During development, collective cell movement is required for tissue morphogenesis and gastrulation, while individual cell migration is observed during primordial germ cell movement^1,2^. In adult organisms, cell migration is needed for wound healing, movement of immune and platelet cells^3,4^, and neuronal migration^5,6^. Understanding the underlying mechanisms of cell migration is also crucial in elucidating how certain disease states operate, such as metastatic cancer and autoimmune diseases^6,7^.

Although actin arrays are regarded as the main cytoskeletal regulator of cell migration due to the propelling and contracting forces they exert, microtubules are becoming increasingly more recognized as important players in many aspects of cell migration including cell polarity, lamellipodia formation, focal adhesion turnover, and trailing-edge contractility^8–10^. The multifunctionality of microtubules in regulating cell migration is attributed to their ability to undergo dynamic instability at their plus ends and to organize differing rates of dynamic instability asymmetrically across the migrating cell: microtubules near the leading edge display relatively frequent bouts of growth, while microtubules towards the trailing-edge show much higher rate of instability and shrinkage^8,11^. Because of the importance of cell migration in exacerbating cancer severity via metastasis, there is growing interest in developing therapeutics targeting cell locomotion^12,13^. Although proteins linked to actin polymerization and organization are considered potential targets given their central position in cell migration, microtubule-targeting agents have also been acknowledged as promising anti-migration therapeutics due to their advantage of potentially targeting both cell migration in interphase and cell proliferation via mitotic microtubules^14^.

Here we report that Factor Quinolinone Inhibitor 1 (FQI1), the initial lead of a first-in-class set of novel anti-cancer agents, destabilizes the microtubule network in interphase cells and impairs cell movement. FQI1 has previously been identified as a potent and specific inhibitor of the transcription factor LSF^15^ (encoded by *TFCP2*) and has demonstrated promising efficacy against hepatocellular carcinoma in mouse tumor models^16^. In testing FQIs in animal tumor models of hepatocellular carcinoma, whereas tumor proliferation has been inhibited, no toxicity has been observed as yet at these doses^15,16^. This suggests the phenotype of oncogene addiction, in which only cells in which the oncogenic protein is overactivated are sensitive to its inhibition, but normal cells are less sensitive and survive under the same circumstances. This underscores the need to understand not only how cancer cells respond to FQIs, but also how normal cells respond. Exploring the mechanism behind FQI1’s anti-proliferative activity in cancer cells revealed that FQI1 causes mitotic arrest with disrupted spindles and condensed, but unaligned chromosomes in vivo, as does siRNA specifically targeting LSF^17,18^, which can result in apoptosis or senescence^15,17^. Because FQI1 treatment disrupts mitotic microtubules and LSF also directly and specifically interacts with α-tubulin^19^, we hypothesized that FQI1 would also impact microtubule-associated processes beyond mitosis. Herein we describe the effects of FQI1 on microtubules and cell movement in interphase telomerase-immortalized cells: (1) microtubules are rapidly destabilized in human fetal hepatocytes (FH-B cells) and retinal pigment epithelial (RPE) cells upon FQI1 addition; (2) FQI1 triggers rapid cell compaction in FH-B cells and renders RPE cells more circular in shape; and (3) FH-B and RPE cells exhibit defective cell motility in the presence of FQI1.

## Results

### Interphase microtubules are rapidly depleted in the presence of FQI1

LSF, although initially described as a transcription factor, also directly binds α-tubulin^19^. Furthermore, treatment with the LSF inhibitor FQI1 results in mitotic arrest in multiple cell types, at least in part by disruption of spindle microtubules^18^. To investigate whether FQI1 also disrupts microtubules in interphase, we utilized two cell lines that were immortalized by stable expression of telomerase. First, the absence of detectable liver toxicity in mice dosed with FQIs, either morphologically or by measurement of liver enzymes^15,16^, prompted use of the human fetal hepatocyte line (FH-B) to investigate consequences of FQIs in these non-cancer cells. Second, the human retinal epithelial line (RPE) is often utilized to study normal cytoskeletal behavior, making them excellent to probe. Microtubule destabilization was assessed by microtubule sedimentation assays. Cells were treated with FQI1 for increasing lengths of time, followed by immediate cell lysis in a buffer containing taxol to stabilize pre-existing microtubules, but no other inhibitors. The cellular tubulin pool was then separated by sedimentation into soluble and pellet fractions containing free tubulin and microtubule-associated tubulin pools, respectively^20^.

**Figure 1:**
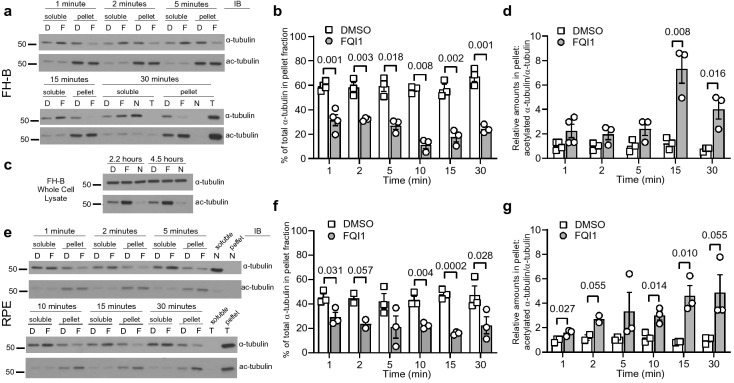
Stable microtubule levels are rapidly diminished, but also acetylated, upon FQI1 treatment. (**a,e**) Representative immunoblots from lysates harvested at the indicated timepoints, derived from FH-B (**a**) and RPE cells (**e**) treated with 4 µM FQI1 (“F”) or vehicle (“D”, 0.01% DMSO) and separated into soluble and insoluble tubulin fractions using a microtubule sedimentation assay. Blots were probed for α-tubulin (upper blots), then reprobed for K40 acetyl- α-tubulin (lower blots). Microtubule-stabilizing taxol (“T”, 1 µM) and microtubule-destabilizing nocodazole (“N”, 1 µM) were added to the cells for 30 min and served as positive and negative controls, respectively. (**b,f**) Quantitation of the percentage of the total α-tubulin in the insoluble fractions at the indicated time-points after addition of FQI1 or vehicle in FH-B cells (**b**) and RPE cells (**f**). Three to four independent experiments were performed for each cell line; *p* values were generated using an unpaired two-sample t-test. Bars and corresponding error bars represent mean ± s.e.m. Numbers above brackets represent *p*-values. (**c**) FH-B cells were pretreated with 2 mM thymidine for 18 h, followed by incubation in fresh media containing 2 mM thymidine and either 4 µM FQI1, 1 µM nocodazole, or vehicle (0.01% DMSO) for the indicated times. Whole cell lysates were blotted for ⍺-tubulin and acetyl-⍺-tubulin. This experiment was performed once. (**d,g**) Quantitation of the amount of insoluble acetylated α-tubulin relative to the total insoluble α-tubulin at the indicated time-points after addition of FQI1 or vehicle in FH-B cells (**d**) and RPE cells (**g**). *P*-values were calculated using an unpaired two-sample t-test. Bars and error bars represent the mean ± s.e.m. from 2 to 4 biological replicates. Numbers above brackets represent *p*-values. For full immunoblots of (**a**,**c**,**e**), see Supplementary Fig. S7 online.

The time-course data demonstrated not only that FQI1 was effective at reducing stable microtubule levels in FH-B cells, but also that this decrease in microtubules occurred extremely quickly, as a significant drop in pelleted tubulin was observed only 1 min after FQI1 (F) addition (Fig. 1a,b). As positive and negative controls, taxol (T) increased the level of polymerized tubulin and nocodazole (N) decreased the level of polymerized tubulin, as shown after incubation of cells with these compounds for 30 min (Fig. 1a). The decrease in stable microtubules persisted for at least 1 h in the presence of FQI1, but then was reversible approximately to control levels within 1 h after removal of FQI1 (see Supplementary Fig. S1 online).

Total α-tubulin levels remained constant in cells, even with longer exposure to FQI1 (Fig. 1c). Unexpectedly, however, the total levels of acetylated α-tubulin were significantly increased after FQI1 treatment, in contrast to the decrease after nocodazole treatment that was expected (Fig. 1c). Examination by the microtubule sedimentation assay confirmed that the FQI1-induced microtubule destabilization was accompanied shortly thereafter by an increase in the amount of acetylated α-tubulin in the microtubule fraction. This occurred despite the significant, but never complete, depletion of microtubules (Fig. 1a,d, 15- and 30-min pellets). Both the kinetics of microtubule disruption and of enhanced α-tubulin acetylation in the microtubules were similar in RPE cells treated with FQI1 (Fig. 1e–g). This combination of microtubule acetylation and destabilization is distinct from the effects observed by nocodazole, which shifts the tubulin pool to the depolymerized state and results in loss of α-tubulin acetylation overall, and taxol, which stabilizes microtubules, enhancing the population of longer-lived microtubules that tend to be more acetylated (Fig. 1a,b, ”N” and “T” lanes).

It has been reported that the complete collapse of microtubules as a consequence of nocodazole treatment can involve, in part, a mechanical compression of nocodazole-resistant microtubules by myosin-mediated contraction resulting from RhoA activation^21^. Although treatment with FQI1, unlike nocodazole, does not lead to a complete collapse of microtubules, we nonetheless tested whether inhibiting the phosphorylation of myosin light chain 2 (MLC2), which activates myosin II-mediated contraction, could rescue FQI1-mediated microtubule depletion. However, FH-B and RPE cells pre-exposed to Y-27632 at a concentration sufficient to inhibit MLC2 phosphorylation experienced quantitatively the same decline in stable microtubules as seen in control cells (see Supplementary Fig. S1 online). Overall, these data demonstrate that FQI1 significantly and rapidly decreases the pool of stable microtubules in interphase in these two cell types.

**Figure 2:**
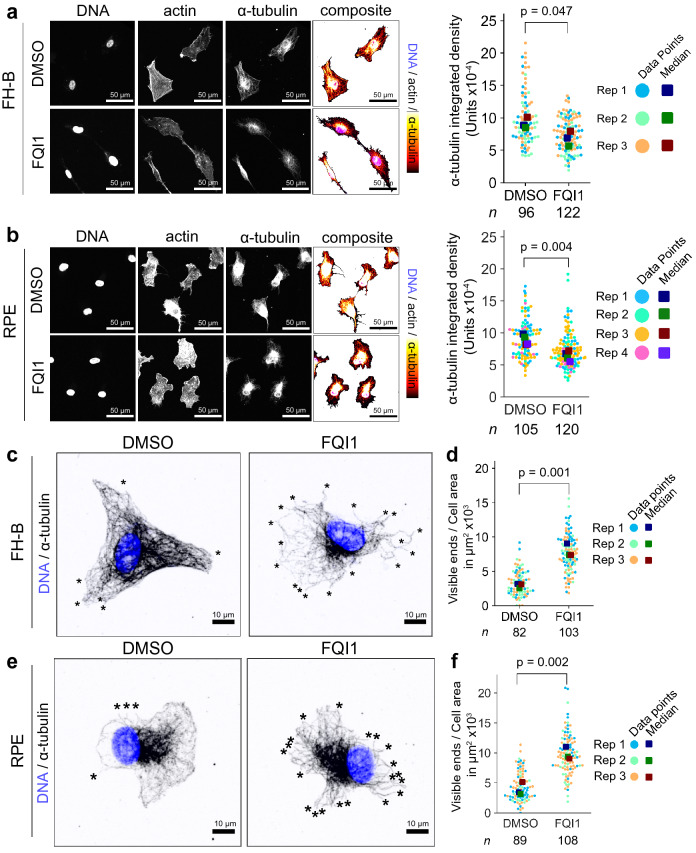
Total tubulin density decreases and visible microtubule ends increase with FQI1 treatment. FH-B (**a**) and RPE (**b**) cells were pretreated with 10 µM Y-27632 for 30 min in order to maintain cell shape and then treated for 30 min (FH-B cells) or 10 min (RPE cells) with either 4 µM FQI1 or vehicle (0.01% DMSO). After fixing, cells were immunostained for α-tubulin, followed by fluorescently labelled Phalloidin and Hoechst 33342 staining. The actin staining was used to determine the cellular space as a region of interest for the measurement of α-tubulin intensity. To generate the composite images, the actin channel was thresholded, inverted and combined with the other channels using ImageJ. To better visualize the α-tubulin staining, the “Red Hot” look-up table was applied to the α-tubulin channel; the white-yellow and red–black colors represent the high and low intensities of the α-tubulin channel, respectively. Purple color represents the overlap of the blue and red colors. *P*-values were calculated using an unpaired two-sample t-test and the integrated density values of all cells from 3–4 independent experiments were incorporated into swarmplots. The total number of cells analyzed in each condition from all independent experiments is indicated by “n”. When calculated in bulk using the Mann–Whitney U test, *p* values were 2.0 × 10^–5^ (**a**) and 8.3 × 10^–7^ (**b**). (**c-f**) Representative fluorescence images of digitally magnified FH-B (**c**) and RPE (**e**) cells are depicted. Visible microtubule ends are marked with asterisks. Total visible microtubule ends in FH-B (**d**) and RPE (**f**) cells were counted in both treatment groups, divided by the cell spreading area, and integrated into swarmplots. Statistical significance was determined by using an unpaired two-sample t-test on medians of the biological replicates. When calculated in bulk using the Mann–Whitney U test, *p* values were < 2.2 × 10^–16^ (**d,f**).

As an independent assay for monitoring the effect of FQI1 on microtubules, the microtubule network was imaged by immunofluorescence. A significant decrease in overall α-tubulin intensity was observed after fixation of both FQI1-treated FH-B and RPE cells, compared to their respective cells treated with vehicle (Fig. 2a,b), consistent with the microtubule sedimentation assay.

A closer look at the microtubule network in FQI1- versus vehicle-treated cells revealed a striking 2.7- to 2.5-fold increase in the number of exposed and individually discernible microtubule ends in the presence of FQI1 in FH-B and RPE cells, respectively (Fig. 2c–f). We interpreted these individually discernible microtubule ends as indicators that the microtubule network was undergoing depolymerization to such an extent that individual microtubule ends were becoming more visible, rather than being embedded in a dense meshwork. Overall, the decrease in stable microtubules, the decrease in total tubulin density and the increase in exposed microtubule ends indicate a rapid and severe FQ11-mediated disruption of microtubules in interphase cells.

### FQI1-mediated microtubule depletion induces rapid compaction in immortalized human fetal hepatocytes

**Figure 3:**
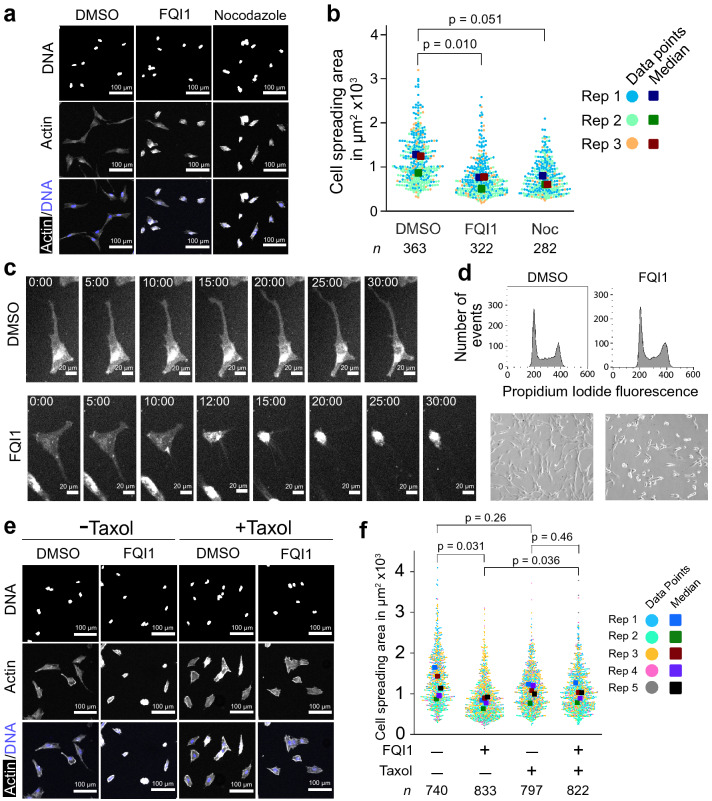
FQI1 treatment induces a rapid cell compaction in FH-B cells. (**a**) Representative fluorescence images after staining for actin and DNA following a 30-min treatment of FH-B cells with 4 µM FQI1, 1 µM nocodazole, or vehicle (0.01% DMSO). (**b**) The areas covered by individual cells, following 30–60 min treatments as depicted in (**a**), were quantified using ImageJ and displayed as swarmplots. *P*-values were calculated using a paired t-test on medians of the biological replicates. The number of cells analyzed in each condition is indicated by “*n*” and was pooled from three independent experiments. When calculated in bulk using the Mann–Whitney U test, *p* values were < 2.2 × 10^–16^ (between DMSO and both FQI1 and Nocodazole). FH-B cells treated with FQI1 for 60 min (replicate 3) received fresh media along with the respective treatment. (**c**) FH-B cells were stained with CellBrite Steady Membrane stain, treated with 4 µM FQI1 or vehicle (0.01% DMSO), and immediately imaged by time-lapse microscopy. Shown are images of representative cells taken at 2–5 min intervals, derived from one biological replicate. Images were taken using a 20 × objective. (**d**) Representative flow cytometry analysis of cellular DNA content showing cell cycle profiles of asynchronous FH-B cells treated with 4 µM FQI1 or vehicle (0.01% DMSO) for 1 h (upper row), along with phase contrast images of cells taken prior to harvesting and fixation (lower row). Cells in this experiment received fresh media along with the respective treatment. Phase contrast images were taken using a 10 × objective on an Olympus IX50 microscope. (**e**), After pretreatment with 1 µM taxol or vehicle (0.01% DMSO) for 30 min, FH-B cells were incubated in either 4 µM FQI1 or vehicle (0.02% DMSO) for another 30 min before fixation. Representative fluorescence images of actin- and DNA-stained cells are shown following a 30-min treatment. (**f**) Swarmplots of cell spreading area were calculated for individual cells incubated with FQI1 or DMSO for 30 min (replicates 1–3) as in (**a**), as well as cells incubated for only 10 min (replicates 4–5), since comparison of these datasets showed that both the spread of the data and the medians were comparable, consistent with compaction occurring within 10–15 min as shown in (**c**). Paired t-tests were performed on the medians between treatment groups from these five replicates. When calculated in bulk using the Mann–Whitney U test, the *p* values for + /− FQI1 were < 2.2 × 10^–16^ in the absence of taxol and 0.388 in the presence of taxol, and the *p* values for + /− taxol were < 2.2 × 10^–16^ in both the absence and presence of FQI1.

Upon investigation of the phenotypic consequences of FQI1 treatment, when asynchronous FH-B cells were treated for 30 or more minutes with FQI1, the cells underwent a notable compaction, as visualized by staining for actin (Fig. 3a). Quantification revealed statistically significant, 1.7-fold reduction in cell spreading area in FQI1-treated cells compared to vehicle-treated cells (Fig. 3b). This decrease in cell spreading area was phenocopied by treatment with nocodazole, which also results in fewer stable microtubules (Fig. 3a,b). This cell compaction phenotype was reversible, even after a 6-h treatment with FQI1 (see Supplementary Fig. S2 online).

As monitored by time-lapse microscopy, the overall spreading of FH-B cells was substantially reduced within 10 min after addition of FQI1, accompanied by a retreat of cellular protrusions between 10 and 15 min (Fig. 3c). In addition, cell blebbing was observed in the presence of FQI1 (see Supplementary Movies 1 and 2), which is consistent with activation of the myosin II pathway via the observed phosphorylation of MLC2. Since FQI1 can cause cells to undergo mitotic arrest^17,18^, which also results in cells “rounding-up”, although not as quickly, the cell cycle stage of cells exhibiting this compact phenotype was examined by cellular DNA profiling. By this analysis, there was no apparent shift in cell cycle phases upon such a short incubation with FQI1 (Fig. 3d). Furthermore, when FH-B cells were synchronized by a single thymidine block, which causes cells to arrest around the G_1_/S transition and into early S-phase, cells treated with FQI1 displayed the same change in morphology as did asynchronous FH-B cells (see Supplementary Fig. S2 online), confirming that this FQI1-induced phenotype is the consequence of a non-mitotic action.

Because the decline in stable microtubules temporally preceded the FQI1-induced reduction in cell spreading in FH-B cells, we tested whether the morphological changes were connected to the prior depletion of microtubules. In particular, we investigated whether taxol, a microtubule-stabilizing agent, would rescue the cell compaction of FH-B cells resulting from exposure to FQI1. Incubating taxol with FQI1 fully inhibited the reduction in the level of stable microtubules, as determined by the microtubule sedimentation assay (see Supplementary Fig. S2 online). Indeed, when FH-B cells were incubated first with taxol, subsequent FQI1 treatment did not visibly lead to the compressed phenotype (Fig. 3e). Upon quantitation, whereas the average median cell spreading area upon incubation with FQI1 alone was decreased 1.5-fold (Fig. 3f), similarly to previous experiments (Fig. 3b), there was essentially no detectable effect of FQI1 on cell spreading area when cells were pretreated with taxol (an average median decrease of 1.05-fold). In the absence of FQI1, although the swarmplot of adding taxol alone appeared to shift the cell spreading area (a 1.15-fold decrease in the average median area), this was not statistically different from the control (*p* = 0.26; Fig. 3f). Results obtained from the taxol rescue experiment after either a 10-min or 30-min incubation with FQI1 were comparable, as both the means and spread of the cell spreading areas overlapped (Fig. 3f); this is consistent with cell compaction being completed within a 10–15-min time frame (Fig. 3c). Taken together, the ability of taxol to block FQI1-mediated cell compaction support the interpretation that FQI1-induced reduction in cell spreading is mediated through destabilization of microtubules.

In considering whether the rapid microtubule disruption resulting from FQI1 treatment could trigger cell contraction, we examined the phosphorylation status of myosin light chain 2, which when phosphorylated on serine 19 activates of myosin II-mediated contraction. Indeed, phosphorylation of MLC2 was elevated 10-min after FQI1 addition (see Supplementary Figs. S1 and S2 online). Furthermore, the increase in MLC2 phosphorylation was abrogated when FH-B cells were preincubated with taxol, strongly suggesting that FQI1-mediated microtubule depletion is needed in order to activate the myosin II pathway. Both ROCK and MLCK can directly phosphorylate MLC2 at serine 19, although only ROCK also phosphorylates, and thereby inactivates, the phosphatase (MLCP) that dephosphorylates serine 19 on MLC2. Thus, by inactivating ROCK, both direct ROCK-mediated phosphorylation of MLC2 is inhibited, but also phosphorylation by other kinases (e.g. MLCK) of MLC2 at that site is also inhibited. Thus, to explore whether FQI1-facilitated compaction of FH-B cells is dependent on the pathways that converge on phosphorylated MLC2 (pMLC2), we tested whether the ROCK inhibitor Y-27632 would counteract the ability of FQI1 to reduce the cell spreading area. To this end, cells were pretreated with either Y-27632 or vehicle, followed by addition of FQI1 or vehicle for an additional 30-min (see Supplementary Fig. S3 online). FQI1 still caused a statistically significant decrease in cell spreading area in the presence of Y-27632, although the extent of reduction in cell spreading area when cells were pretreated with Y-27632 (1.2-fold) was slightly diminished compared to the control group (1.6-fold) when comparing the medians. This suggests that the myosin II pathway, although activated by FQI1 in a microtubule-dependent manner, is surprisingly only a partial, but not the sole mediator of FQI1-induced cell compaction, and that factors other than the ROCK pathway can enable FQI1’s control over cell shape. In order to address this issue more directly, a rescue experiment was also performed using blebbistatin to inhibit the myosin II pathway. The results generally mirrored those obtained with Y-27632. The extent of reduction in cell spreading area when cells were pretreated with blebbistatin (1.5-fold) was only slightly diminished compared to the control group (1.6-fold) when comparing averages of the medians. Thus, there was only a trend toward reduction of cell spreading in the presence of blebbistatin (see Supplementary Fig. S3 online). Overall, these data indicate that FQI1 activates the myosin II pathway by directly or indirectly depolymerizing microtubules in FH-B cells, but that this pathway is only partially involved in the FQI1-induced cell compaction.

### Immortalized retinal pigmented epithelial cells become more circular with FQI1 treatment

**Figure 4:**
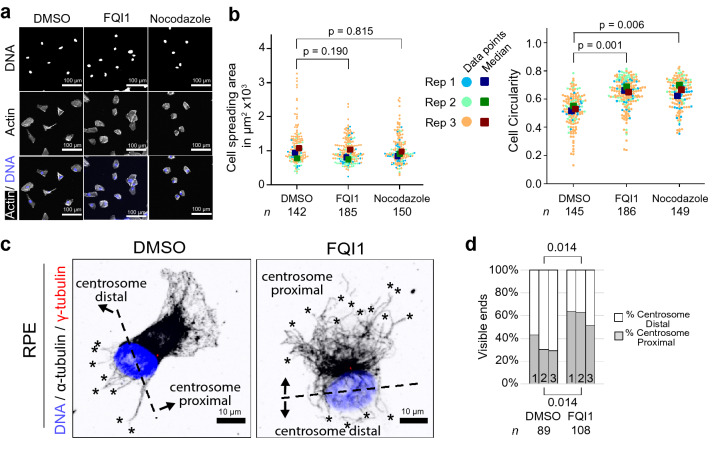
FQI1 treatment increases circularity in RPE cells. (**a**) Representative fluorescence images of RPE cells after 1-h treatment with 4 µM FQI1, 1 µM nocodazole, or vehicle (0.01% DMSO). Cells received fresh media along with the respective treatment. (**b**) Cell spreading area (left panel) and circularity (right panel) of RPE cells was quantified by processing fluorescent images of cells as in (**a**) in ImageJ. Swarmplots represent all analyzed cells from three independent experiments; *p*-values were calculated using a paired two-sample t-test. The number of cells analyzed in each condition is indicated by “*n*”. When calculated in bulk using the Mann–Whitney U test, *p* values for the cell spreading area comparisons were not statistically significant (0.085 for DMSO/FQI1 and 0.29 for DMSO/nocodazole), and for the circularity analysis were < 2.2 × 10^–16^ and 3.0 × 10^–16^ for the DMSO/FQI1 and the DMSO/Nocodazole comparisons, respectively. (**c,d**) Representative fluorescence images of digitally magnified RPE cells (**c**) are depicted. Dashed lines separating centrosome proximal and centrosome distal portions of the cells were generated using γ-tubulin and Hoechst localization as guides. Visible microtubule ends, marked with asterisks, were counted as in Fig. 2d,f, and partitioned into percentages in the two sectors, which were averaged and plotted as stacked bar charts (**d**). *P*-values for the centrosome distal and centrosome proximal percentages are indicated above and below the brackets, respectively and were determined using an unpaired two-sample t-test comparing the averages for each group; numbers in stacked bar plots represent the indicated biological replicates as designated in Fig. 2f.

In order to determine whether the FQI1-induced cell shape change in interphase occurs across multiple cell types, we tested whether the phenotype could be replicated in a different cell line. RPE cells are frequently used as a model cell line for investigating cell cytoskeleton and cell migration. In contrast to FH-B cells, RPE cells did not respond to FQI1 with significant change in cell spreading area. However, the RPE cells became significantly more circular in shape (Fig. 4a,b) in the presence of either FQI1 or nocodazole as compared to control cells.

This increase in circularity suggested that RPE cells may be losing their cytoskeletal polarization^22,23^. To test whether microtubule disruption was affected differentially across the RPE cells, we used the increased frequency of visible microtubule ends as markers for microtubule depletion. In order to identify a cellular axis of the RPE cell population, the nucleus and γ-tubulin, marking the centrosome, were stained (Fig. 4c,d). The position of the centrosome relative to the nucleus is often an indicator for the anterior leading edge of a migrating cell^24^. Although this positioning can vary with cell type and substrate, in unidirectionally migrating RPE cells the centrosome is predominantly anterior, independent of the migratory mode^25^. In a population of sparse cells, in which directionality can switch, this would not necessarily be the case, although under our 2-dimensional tissue culture conditions, even sparse RPE cells, when untreated, migrate overall in a single trajectory. Analyzing only cells containing a strongly stained single location of γ-tubulin, and using the centrosome positioning as a marker of different cell sectors, 60–70% of visible microtubule ends in control RPE cells occurred at the centrosome distal edge, while microtubules at the centrosome proximal edge formed a meshwork in which individual microtubule ends were much less discernible (Fig. 4c,d). This is consistent with a posterior/anterior assignment, as microtubules undergoing higher rates of catastrophes at the trailing edge, in which the local microtubule network is depleted^26,27^, would be anticipated to render individual microtubules more visible. In contrast, visible microtubule ends in FQI1-treated RPE cells were more equally distributed relative to the nuclear-centrosomal positioning in the cell (Fig. 4c,d). This is consistent with the increase in circularity observed in FQI1-treated RPE cells, because a more circular shape could indicate a loss in cell polarization, which depletion of microtubules towards the leading portion of the cells would reflect.

In the case of FH-B cells, the centrosome distal–proximal cellular distribution of visible microtubule ends was even in control cells, and largely unaffected by FQI1 (see Supplementary Fig. S4 online). The morphological response to FQI1 of FH-B cells, overall cell compaction, is consistent with such an even depletion of microtubules throughout the cell. Overall, these data suggest, although do not prove, that the FQI1-mediated decrease in microtubule levels in RPE cells is accompanied by a reduction in microtubule polarization, which may result in the increase in cell circularity.

### FQI1 impairs cell motility

**Figure 5:**
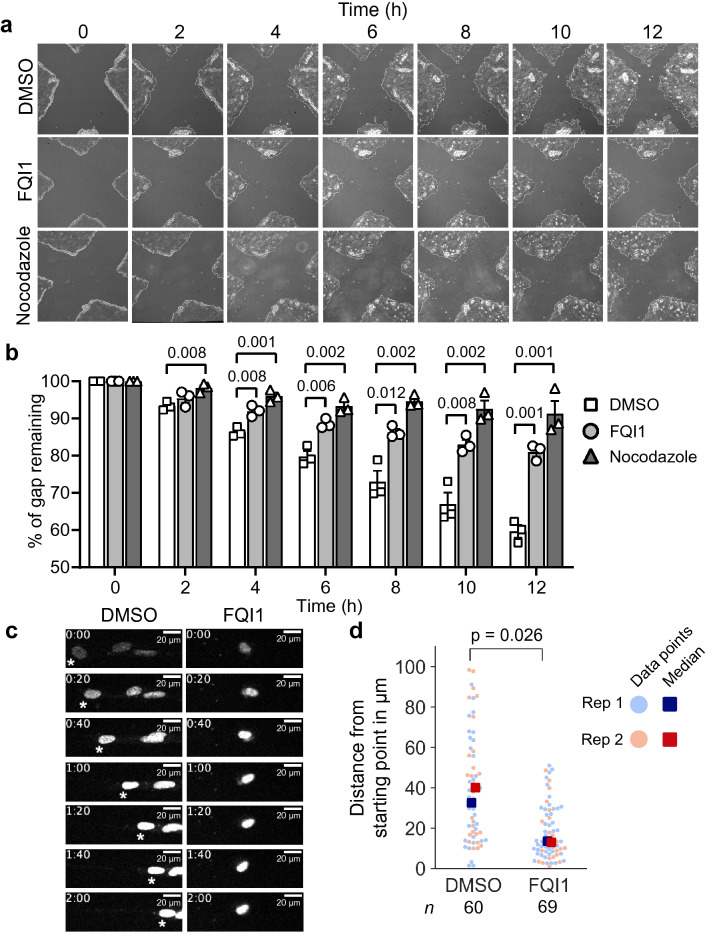
FQI1 impairs wound healing ability and cell motility. (**a,b**) After confluent RPE cells were pretreated overnight with 2 mM thymidine, an X-shaped wound was drawn into the cell monolayer. The cells were monitored for 12 h by phase contrast imaging using a 4 × objective following treatment with 4 µM FQI1, 1 µM nocodazole, or vehicle (0.01% DMSO). (**a**) Representative phase contrast images of the X-shaped wound from each treatment group over the 12-h time-course are shown. (**b**) Quantitation of the averaged distances between the wound’s four vertices normalized to the 0-h distance are shown. Bars and error bars represent the mean ± s.e.m. The scale of the y-axis is presented from 50 to 100%, to expand the relevant portion of the graphs. Three independent experiments were performed. Statistical significance was tested using an unpaired two-sample t-test. Numbers above brackets represent *p*-values. (**c,d**) FH-B cells pretreated overnight with 2 mM thymidine were treated with 4 µM FQI1 or vehicle (0.01% DMSO) for 1 h. Cells were then stained with NucSpot Live 650 dye and monitored by time-lapse fluorescence microscopy for 2 h. The distances travelled from starting point to end point were quantified and pooled together from two separate experiments to generate swarmplots. Images of representative nuclei of migrating FH-B cells from both treatment groups, taken at 20-min intervals, are shown in (**c**). Note that nuclei increase in intensity with time due to accumulation of the stain. Timestamps denote hours:minutes and the asterisks in the left panel indicate the path of the relevant, representative cell. For the entire population of cells over the time course, see Supplementary Movies 3 and 4. The swarmplot of distances travelled by the FH-B cell population is shown in (**d**). Statistical significance was tested by an unpaired two-sample t-test on the means of two biological replicates. When calculated in bulk using the Mann–Whitney U test, the *p* value was 7.5 × 10^–7^ between DMSO and FQI1 treatments. The total number of cells analyzed in each condition is indicated by “*n*”.

Concentrations of nocodazole that result in overall depolymerization of the microtubule network also suppress directional cell migration^11,28^. Because nocodazole mimics the morphological phenotypes of FQI1-treated FH-B and RPE cells, we tested whether FQI1 would impede cell migration. This was investigated using a wound-healing assay in RPE cells, which, in contrast to the fibroblast-like FH-B cells, are able to form stable monolayer sheets. In order to remove cell proliferation as a significant variable from these experiments, since both FQI1 and nocodazole are well established to prevent completion of mitosis at these concentrations, cells were preincubated with 2 mM thymidine overnight, with replenishment of the thymidine immediately prior to the wound healing assay. This high concentration of thymidine depletes the nucleotide pools, thereby limiting cell cycle progression past the G1/S transition or early S phase. Analysis of the cell cycle profiles of the treatment groups at the end of the 12-h experiment showed only ~ 10% more cells with G2/M-content of DNA in the presence of FQI1 or nocodazole compared to controls, indicating that maximally ~ 10% of the cells in the populations slipped through the cell cycle block during the course of the experiment (see Supplementary Fig. S5 online). At 0 h, an X-shaped scratch was drawn on confluent monolayers of cells and the degree of wound-closure under different treatment conditions was monitored every 2 h. FQI1 significantly hindered RPE cells from migrating into the wound, similarly to nocodazole (Fig. 5a,b), as quickly as the 4-h timepoint. This period of time is too short to represent a proliferative defect. By 12 h, whereas the control cells had migrated into 40% of the gap, the FQI1-treated cells had only migrated into 19% of the gap. Overall, these results indicate that FQI1 inhibits cell migration.

The migratory behaviors of both FH-B and RPE cells in the presence or absence of FQI1 were directly analyzed by tracking the paths of individual cells using time-lapse microscopy. This experiment was also performed in cells arrested around G1/S by a thymidine block, to avoid significant concerns regarding cell cycle effects of FQI1. Furthermore, any individual cells that did undergo cell rounding or cell division were discounted during the analysis. Cells were treated with FQI1 or vehicle for 1 h and then stained with a live-cell nuclear dye for cell tracking, while maintaining cells in either FQI1 or vehicle treatment. Measuring the cell displacement over a 2-h period from starting point to end point demonstrated that FQI1-treated FH-B cells display a significantly smaller range of motion than do control cells (Fig. 5c,d; see Supplementary Movies 3 and 4). In RPE cells, there was also a clear trend towards motility being reduced in the presence of FQI1, although it did not reach the level of statistical significance (see Supplementary Fig. S5 online). This is consistent with the results from the wound healing assay performed with RPE cells, in which the difference in cell movement between FQI1- and control-treated cells was not statistically significant after 2 h. The extent of FQI1-mediated changes to cell locomotion also appear to be cell type dependent, as the movement of FH-B cells was more oscillatory and stochastic in the presence of FQI1 (Fig. 5c, and Supplementary Movies 3, 4 online), while the majority of RPE cells were simply decelerated by FQI1 (see Supplementary Fig. S5 and Movies 5, 6 online). Overall, the data indicate that FQI1 treatment diminishes cell motility.

### Cell compaction and inhibition of cell migration scale with compound efficacy of FQIs

To test whether the novel phenotypic effects in FH-B and RPE cells are related to the previously described activities of FQI1^15,17^, we tested whether these phenotypes were replicated using a recently developed FQI family member, FQI2-34. FQI2-34 is more potent than FQI1, exhibiting 17-fold higher efficacy than FQI1 in inhibiting proliferation of FH-B cells (see Supplementary Fig. S6 online). Cell spreading of FH-B cells was decreased 2.1-fold by FQI2-34 at a 20-fold lower concentration than the effective concentration for FQI1 (see Supplementary Fig. S6 online). In RPE cells, FQI2-34 is similarly more potent than FQI1, exhibiting 16-fold higher efficacy than FQI1 in inhibiting proliferation (see Supplementary Fig. S6 online). In the wound healing assay, a 20-fold lower concentration than the effective concentration for FQI1 almost completely blocked cell migration, showing a statistically significant effect even at 2 h after wounding. This was not clearly not a proliferative consequence, not only due to the incubation with thymidine to inhibit cell cycle progression, but also because the effect occurred within only 2 h. Therefore, both the rapid morphological change in interphase FH-B cells and the inhibition of cell migration of interphase RPE cells are likely due to disruption of similar cellular target(s) specific to FQI1 that lead to mitotic defects in other cell types, such as cancer cells. Notably, in cell extract thermal shift assays (CETSA)^29,30^, FQI2-34, like FQI1, enhanced the stability of LSF to thermal denaturation, indicating that both compounds directly bind LSF (see Supplementary Fig. S6 online). These data are consistent with the involvement of LSF as the target of FQIs resulting in the changes in cell shape and migration, although other mechanisms remain possible.

## Discussion

FQI1 is an anti-proliferative compound, which has demonstrated promise in curbing cancer cell growth in vitro and in vivo^15,16^. Recent investigations showed that FQI1 causes mitotic arrest with condensed, but unaligned chromosomes by disrupting microtubule spindles^17,18^. Here, we present the significant cytoskeletal and morphological impact of FQI1 also on interphase cells, due to disruption of microtubules and microtubule-associated processes. Specifically, we demonstrate first that FQI1 induced cells to undergo rapid microtubule depolymerization in two different cell lines, with fractionation assays demonstrating a widespread, although not complete, reduction in the stable microtubule pool by 1 min after FQI1 addition. Imaging revealed disrupted microtubule networks, in that free microtubule ends were increased significantly. Morphologically, in immortalized human fetal hepatocytes (FH-B cells), microtubule disruption is followed by cell compaction, which occurred around 10–15 min after addition of FQI1. The microtubule stabilizer taxol prevented FQI1-mediated cell compaction, signifying that microtubule depolymerization drives this cellular compression. Both the microtubule disruption and the cell morphology changes were reversible, when FQI1 was washed out of the medium, further suggesting that the two processes are linked and specific. The immortalized retinal pigment epithelium (RPE) cells instead responded to FQI1 with increased cellular circularity. In addition, whereas the majority of visible microtubule ends were highly skewed in control cells, to the side of the nucleus opposite from the bulk of the microtubule network, they were more equally distributed across the cytoskeletal axis after treatment with FQI1; this may implicate FQI1 in perturbing cell polarization. Finally, FQI1 treatment diminished the ability of RPE cells to close a wound gap and overall reduces the rate of FH-B and RPE cells’ movement in sparsely seeded populations. Taken together, our findings support a role for FQI1 in disrupting interphase microtubules, which reduces cell spreading and overall impedes cell motility.

Although the cell morphology effects of FQI1 on the two cell lines seem quite distinct, it is striking that both the swift FQI1-mediated reduction in cell spreading in FH-B cells and the more rounded morphology in RPE cells are similar to morphological changes caused by nocodazole in these cell types (Figs. 3a,b, 4a,b). It is widely viewed that nocodazole has a single target—it binds free β-tubulin and inhibits tubulin polymerization, thereby resulting in the loss of microtubules. This suggests that microtubule defects can indeed be a common basis resulting both in cell compaction of FH-B cells and enhanced circularity of RPE cells. Thus, despite the presumed mechanistic differences in how nocodazole and FQI1 induce microtubule depolymerization, the gross phenotypic outcomes are nonetheless similar.

How microtubules are mechanistically destabilized upon exposure of cells to FQI1 is not yet defined. An insight is provided by the enhanced microtubule acetylation, a post-translational modification of α-tubulin located inside the microtubule lumen, which is generally associated with long-lived and stable microtubules^31^. The combination of depolymerization with hyperacetylation of remaining microtubules sets FQI1 apart from the microtubule targeting agents shown here: nocodazole resulted in a complete loss of microtubules, concomitantly resulting in tubulin deacetylation, while taxol stabilized microtubules, resulting in hyperacetylated tubulin. However, vinblastine can both disrupt the structure of microtubules, but nonetheless enhance acetylation of the remaining microtubules. This is likely due its idiosyncratic conformational restructuring of protofilaments to generate spiral aggregates, which then allows greater access to residues in the lumen^32,33^. Multiple mechanisms have been proposed for the tubulin acetyltransferase, αTAT1, to access the microtubule lumen to acetylate α-tubulin. Either open microtubule ends or gaps in the polymer lattice due to mechanical damage or bending can provide entry points for αTAT1^21,34–36^. In the case of FQI1, the increase in visible microtubule ends at the cellular periphery suggests that αTAT1 may enter the microtubule lumen mainly through open polymer ends. On the other hand, the significant increase in acetylation of the microtubule structures leaves open the possibility that FQI1 treatment might either directly or indirectly mechanically injure or alter the conformation of the microtubule lattice. Additional studies are required to distinguish among these possibilities.

In investigating how FQI1-mediated microtubule disruption resulted in cell compaction in FH-B cells, we considered the established mechanism for nocodazole-mediated contraction in which depolymerization of microtubules results in dissociation of GEF-H1 from microtubules, which then activates the small GTPase RhoA^8,28,37,38^. Subsequently, ROCK, an effector protein of RhoA, targets several downstream proteins for phosphorylation including MLC2 in order to execute the contractile response^39,40^. As anticipated, MLC was phosphorylated in FQI1-treated cells during the same time frame in which cells were undergoing compaction. However, surprisingly, neither prevention of MLC2 phosphorylation by a ROCK inhibitor nor more directly targeting myosin II activity with blebbistatin were sufficient to fully block FQI1-mediated cell compaction. This suggests that FQI1 may influence the actin cytoskeleton through multiple pathways.

In proposing mechanisms both for how FQI1 impacts microtubule stability and its acetylation and for how FQI1 impacts cell morphology, a crucial aspect is whether or not it involves FQI1’s intended target protein, the transcription factor LSF (TFCP2). FQI1 was originally identified as a lead compound capable of inhibiting LSF’s ability to transactivate target genes. Given that elevated LSF expression can promote oncogenesis, such as in hepatocellular carcinoma^15,41^, these small molecule LSF inhibitors were viewed as having potential for development of cancer chemotherapeutics. An increasing number of cancer types are being identified in which LSF is overexpressed and contributes to tumorigenic phenotypes^42–44^. The mechanism of action through which FQI1 inhibits proliferation of cancer cells in culture has uncovered disruption of mitotic microtubules and induction of a mitotic arrest^16–18^. Although the possibility that FQI1 exerts anti-mitotic activity through off-target effects has not been ruled out, several lines of evidence point towards LSF being the specific target of FQI1. Notably, knocking down LSF via siRNA generated a similar mitotic arrest to that of FQI1^17^. Furthermore, the IC_50_ of FQI1-mediated inhibition of LSF linearly correlates with the GI_50_ of FQI1-induced cell growth inhibition^45^.

One key set of observations in understanding microtubule effects of LSF inhibitors is that LSF directly binds α-tubulin, can enhance methylation of α-tubulin via the methylase SET8^19^, and can enhance tubulin polymerization in vitro^46^, all suggesting that LSF is a microtubule-associated protein. FQI1 not only inhibits the DNA-binding activity of LSF^15^, which requires oligomerization of the LSF stable dimers to homo-tetramers, but also inhibits interaction of LSF with other specific protein partners^47^. Of particular relevance here, mass spectrometry analysis of LSF interacting proteins in mitotic cell lysates showed FQI1-sensitive associations between LSF and a number of microtubule-associated proteins that are involved in spindle assembly an d dynamics^18^. Given these microtubule-associated properties of LSF, we propose that one biological role for LSF is to promote and/or stabilize the microtubule network, and that binding of FQIs to LSF interferes with that activity. A corollary of this model that could explain distinct overall microtubule-related phenotypes of FQIs in different cell types (e.g. compaction in FH-B cells versus increased circularity in RPE cells) would be differential expression of FQI1-sensitive LSF partner proteins involved in microtubule structure and dynamics.

In interphase, although LSF is predominantly localized in the nucleus, as consistent with its role as a transcription factor, LSF is also localized in the cytoplasm, with levels varying in a cell-type and condition-dependent manner^48–50^. With regards specifically to the effects of FQIs on interphase microtubules, we demonstrate here that the more efficacious FQI2-34, which also binds LSF, induces cell compaction in FH-B cells at concentrations 20-fold lower than the effective FQI1 concentrations (see Supplementary Fig. S6 online). Because the concentration of FQI2-34 that alters interphase cell shape is comparable to the concentration needed to inhibit cell growth, this is consistent with LSF being the mediator of the effects of the compound on interphase microtubules. In addition to the cell compaction phenotype, blebbing was observed in FQI1-treated cells in the time lapse microscopy. We previously noted similar blebbing behavior in both FQI1- and LSF siRNA-treated HeLa cells during studies on mitotic consequences^17^. Since blebbing is associated with myosin II activity, the ability of siRNA specific to LSF to also induce such cell behavior suggests that inhibiting other LSF-related mechanisms can impact the actin cytoskeleton. One possibility is that LSF directly interacts with and affects proteins regulating the actin cytoskeleton network.

The effects of FQI1 on cell motility and migration are also relatively rapid—within 2 h of treatment with FQI2-34 in the wound healing assay. We therefore hypothesize that, if FQI1 effects on motility are a faithful readout of LSF functionality, they are likely due to disruption of dynamic interactions between LSF and partner proteins involved in the cytoskeleton. LSF has been shown in cancer cells to regulate cell migration, invasion, and metastasis, although these functions were reported with elevated levels of LSF transcriptionally upregulating genes important for these processes, using cell lines that stably overexpress LSF^41,49,51,52^. Such slower LSF-mediated transcriptional outputs that occur during oncogenesis, or normal developmental processes could more permanently alter the cellular state. As a complement to the slower process of more permanent changes in cell function, dynamic and rapidly reversible LSF-protein interactions that directly and rapidly alter microtubule stability could achieve similar migration and motility outcomes in a transient manner.

In evaluating FQIs for development for chemotherapeutics, including combinatorial therapies, it is essential to understand not only the consequences of FQI treatments in cancer cells, but also in normal cells. This is the first study to begin to probe such outcomes, revealing consequences to the microtubule network and cell migration. Future studies in animal models are required to determine whether or not cells in normal tissues respond similarly and to illuminate the apparent lack of toxicity upon systemic FQI treatment. In addition, results presented here suggest a novel role for LSF in regulating microtubule stability, which would add LSF to the list of transcription factors that moonlight in directly binding and contributing to proper functioning of structural cellular proteins^53^.

## Methods

### Cell culture

FH-B cells were obtained from Sanjeev Gupta, Albert Einstein College of Medicine^54^ and were cultured in DMEM with 10% FBS at 37 °C in 5% CO_2_. FH-B cells were verified by assessing *ALB* and *CYP2B* expression, and confirming lack of *FOXA2* expression. The RPE-hTERT Flp-In cell line^55^ from Patrick Meraldi, Université de Genève), called RPE cells here, was cultured in HyClone DMEM with 10% FBS at 37 °C in 5% CO_2_ and had been cultured periodically in 400 μg/mL zeocin to ensure maintenance.

### Inhibitor treatments

FQI1 was synthesized as previously described^15^ and dissolved in anhydrous DMSO. FQI2-34 was synthesized as described in the Supplementary Information online (also Supplementary Fig. S8). FQI1 and DMSO, along with nocodazole, taxol (Sigma Aldrich, T7402), and Y-27632 (Med Chem Express, 129830-38-2), were generally added directly into the cell culture dish from the stock solutions (final DMSO concentration 0.01–0.02%). In indicated experiments, media was changed at the time of adding inhibitors, either (1) to maintain levels of thymidine, and therefore the block to cell cycle progression, in long-term experiments, or initially (2) to add media in which inhibitors were added in advance, thereby ensuring that all cells were treated simultaneously, in order to prevent any treatment time delays due to mixing. The latter point (2) was later shown not to be a concern, as FQI1-mediated effects were generally comparable whether inhibitors were added directly to the cells, or when instead media was refreshed. The concentration of FQI1 that is used, 4 µM, is the approximate concentration at which efficacy in multiple assays plateaus, including cell proliferation assays for both FH-B and RPE cells (see Supplementary Fig. S6 online), and LSF-specific activity assays^15^. For nocodazole, 1 µM was empirically determined to be required for near-complete microtubule depolymerization by 30 min in these cells. For taxol, 1 µM completely stabilized cellular microtubules in both FH-B and RPE cells following a 30-min incubation (Fig. 1a,e), and was therefore added 30 min prior to adding other compounds in rescue experiments.

### Microscopy for cell shape analysis

After seeding overnight in 35 mm, high µ-dishes (ibidi USA Inc., 81,156), cells were treated as indicated in figure legends, washed twice in PBS and incubated in fixation buffer (3.7% formaldehyde in 100 mM PIPES at pH 6.8, 10 mM EGTA, 1 mM magnesium chloride, and 0.2% Triton X-100) for 10 min at room temperature. Cells were washed as before, treated with Phalloidin-iFluor 488 Reagent (abcam, ab176753) for 1 h in 1–5% BSA in PBS, followed by Hoechst 33342 (Thermo Fisher Scientific, H3570) for 5 min. Mounting medium (Fisher Scientific, P10144) and cover slips were applied onto dried cells. Individually discernible cells were imaged on an Olympus Fluoview 10i. Using ImageJ, the phalloidin channel was thresholded and area and circularity values were measured and integrated into swarmplots using Python’s seaborn library.

For time lapse microscopy, FH-B cells in high µ-dishes were incubated with CellBrite Steady Membrane stain (Biotium, 30108-T) for 30–40 min in the environmental chamber of a Nikon C2 Si microscope at 37 °C and 5% CO_2_. Treatments were added to the cells through a syringe to reach final concentrations of 4 µM FQI1 or 0.01% DMSO. Immediately thereafter, a time-lapse series was acquired by imaging every 30 s for 30 min at 20× magnification.

### Cell cycle analysis by flow cytometry

After trypsinization, subsequent steps prior to staining were performed at 4 °C. Cells in PBS were transferred dropwise into ethanol (final concentration of 70%) for overnight incubation. Fixed cells were washed with PBS and then incubated in 50 µg/ml propidium iodide and 10 µg/ml RNase A in PBS for 45 min at room temperature. After passage through a cell strainer, cell fluorescence was measured using a Becton Dickinson FACSCalibur. Ten thousand events were counted per sample. Histogram plots were generated using FlowJo.

### Wound healing analysis

Confluent RPE cells were synchronized by incubating with 2 mM thymidine for 18–20 h. Two intersecting lines were drawn into the confluent cells using a pipette tip. A phase-contrast image was acquired of the intersection point (“0 h” time-point) on an Olympus IX50 inverted microscope, immediately followed by treatment with the respective inhibitor and 2 mM thymidine. Phase-contrast images of the intersection point were acquired every 2 h for 12 h, continuing incubation at 37 °C in 5% CO_2_ in-between time points. Using ImageJ, the distances between each pair of neighboring vertices of the intersection point at each time-point were measured and normalized to the “0 h” sample.

All the samples, including a “0 h” synchronized and an asynchronous sample, were harvested and prepared for flow cytometry, as described above. The proportion of cells in their respective cell cycle phases was determined by applying the Dean–Jett–Fox model in the FlowJo Cell Cycle analysis tool.

### Cell motility tracking

After overnight seeding in high µ-dishes, cells were synchronized by incubating in 2 mM thymidine for 18–20 h. Thereafter, cells were treated with 4 µM FQI1 or vehicle (0.01% DMSO) for 1 h, followed by a 10-min incubation in NucSpot Live 650 dye (Biotium, 40082) following the manufacturer’s instructions. Subsequently, cells were imaged every 2 min for 2 h in an environmental chamber of a Nikon C2 Si microscope at 37 °C and 5% CO_2_ at 20× magnification. Cells that underwent mitosis during the 2-h period were excluded from the analysis. Using the Manual Tracking plugin in ImageJ, tracks of individual nuclei were generated by selecting the center of the nuclei in each frame. The distances between starting and end points were calculated and integrated into swarmplots using Python’s seaborn library.

### Cell lysate preparation

For microtubule sedimentation assays, cells were treated as indicated in the figure legends, washed with PBS and incubated in 250 µl of microtubule sedimentation assay lysis buffer (100 mM PIPES pH 6.8, 2 M glycerol, 2 mM EGTA, 5 mM MgCl_2_, 0.1% Triton X-100) supplemented with 1 µM taxol, protease inhibitor cocktail (1:200, Abcam, ab201111) and phosphatase inhibitor cocktail (1:100, Abcam, ab201115) for 20 min at 37 °C. Two hundred µl of scraped lysate was centrifuged at 21,000× g for 10 min at room temperature. Supernatants were saved as soluble fractions. Pellets were resuspended in 200 µl RIPA buffer (50 mM Tris HCl pH 8.0, 150 mM NaCl, 1% IGEPAL CA630, 0.05% deoxycholic acid) supplemented with 1.05% SDS, protease inhibitor cocktail (1:200) and phosphatase inhibitor cocktail (1:100) and incubated at 100 °C for 15 min. Pellet suspensions were centrifuged as before and supernatants were saved as pellet fractions.

For analysis of phosphorylated MLC2, FH-B cells were treated as indicated in the figure legend, washed with PBS and incubated for 5 min in lysis buffer (50 mM Tris–HCl pH 8.0, 50 mM NaF, 5 mM MgCl_2_, 150 mM NaCl, 1% Triton X-100) supplemented with Tricine Sample Buffer (Bio-rad, 1610739), protease inhibitor cocktail (1:200), phosphatase inhibitor cocktail (1:100), and 1% β-mercaptomethanol. Lysates were incubated at 100 °C for 15 min and centrifuged at 21,000×g for 10 min prior to SDS-PAGE.

### Gel electrophoresis and immunoblotting

Microtubule sedimentation fractions and MLC2 analysis samples were separated by electrophoresis through tris–glycine and tricine polyacrylamide gels, respectively, which were transferred to PVDF membranes. Given the high specificity and affinity of the primary antibodies used, resulting in immunoblots with single bands, membranes were often cut prior to further processing, based on the visible molecular weight markers on the blot. This permitted processing of multiple blots together during incubation with antibodies, washing, and precise exposure times, ensuring accuracy in quantitation across multiple blots within a single experiment. Additional practical advantages included conservation of reagents and film. Membranes with microtubule sedimentation fractions and MLC2 analysis samples were blocked in 5% milk in TBST and in 5% BSA in TBST, respectively. After overnight incubation with primary antibody at 4 °C, the membranes were washed and incubated with secondary antibody for 1 h at room temperature. Primary antibodies included anti- α-tubulin (1:2,000, Thermo Fisher Scientific, PA529444, raised against a proprietary region within amino acid residues 179-435), anti-acetylated tubulin (1:1,000, Sigma-Aldrich, T7451), anti-phospho MYL9 (1:200, Thermo Fisher Scientific, PA517727), and anti-β-actin (1:5,000, Sigma-Aldrich, A1978). Secondary antibodies included Goat anti-Rabbit HRP (Thermo Fisher Scientific, 31460; 1:10,000 for anti-α-tubulin; 1:5,000 for anti-phospho MYL9), and Goat anti-Mouse HRP (Thermo Fisher Scientific, 62-6520; 1:5,000 for anti-acetylated tubulin; 1:10,000–1:20,000 for anti-β-actin). Membranes were placed into Pierce™ ECL Western Blotting Substrate (Thermo Fisher Scientific, 32,106). Autoradiography film was exposed to each membrane for various times to ensure acquisition of non-saturated exposures. Band intensities were quantified from scanned films by densitometry using ImageJ.

For reblotting, membranes were incubated in stripping buffer (200 mM glycine, 0.1% SDS, 1% Tween-20, pH 2.2) twice for 10 min, followed by two 10-min washes in PBS and two 5-min washes in TBST.

### Analysis of microtubule features by immunofluorescence

Because Y-27632 partially diminishes FQI1-induced cell compaction (see Supplementary Fig. S3 online) without significantly affecting microtubule levels (see Supplementary Fig. S1 online), cells were pretreated with Y-27632 in order to maintain overall cellular shape, facilitating capture of structural or positional impact of FQI1 on microtubules. In addition, only cells with cell spreading areas larger than 1000 µm^2^ were considered. Cells were seeded overnight in high µ-dishes, treated as indicated in the figure legends, fixed with formaldehyde as described above, blocked in 5% BSA in PBS and stained overnight at 4 °C with anti-γ-tubulin (1:500, Abcam, ab11316) and anti-α-tubulin (1:500, Fisher Scientific, 62204). Samples were then incubated in goat anti-rabbit Cy5 (1:1000, Invitrogen, A10523), goat anti-mouse Alexa Fluor 546 (1:1000, Thermo Fisher Scientific, A11003), and Phalloidin-iFluor 488 Reagent. Hoechst 33342 and mounting medium were applied, before acquiring Z stack images at 60× magnification on an Olympus Fluoview 10i microscope.

Using ImageJ, binary Z projections of Phalloidin and Hoechst channel stacks were merged with Z projections with average intensity of the α-tubulin channels. The Phalloidin channel was used as the region of interest for measurement of “integrated density” of the total α-tubulin intensity, which was plotted into swarmplots.

To assess distribution of visible microtubule ends, the Hoechst, γ-tubulin, and α-tubulin stacks were converted into Z projections with maximum intensity. The Hoechst and γ-tubulin Z projections were thresholded and all channels were merged. A line was generated between the γ-tubulin signal and the center of the nucleus; a line perpendicular to the first line and running through the center point of the nucleus divided the cell into centrosome distal and centrosome proximal portions. Visible microtubule ends were counted using the Cell Counter plugin, excluding unattached microtubule fragments.

### Statistical analysis

In experiments involving large numbers of n, data points of each biological replicate were plotted with the respective median in swarmplots^56^. Medians were chosen due to the non-normal distributions of these datasets. Due to batch effects, paired two-sample t-tests were used to assess statistical significance in cell spreading area experiments. Otherwise, unpaired two-sample t-tests were used. Outliers were excluded using Grubb’s test (α = 0.05). *P*-values less than 0.05 were regarded as statistically significant. In some cases, t-tests were performed using only two independent datasets, when differences were sufficiently compelling and therefore highly unlikely to have occurred by chance. As an alternate measure of statistical significance, datasets with large n values were also analyzed by combining all values from multiple independent experiments and using the Mann–Whitney U test (see figure legends).

## Supplementary Information

Supplementary Video 1.

Supplementary Video 2.

Supplementary Video 3.

Supplementary Video 4.

Supplementary Video 5.

Supplementary Video 6.

Supplementary Information.

## Data Availability

All the relevant data in the article and supplemental information are available from the corresponding author upon reasonable request.
