# Supplementary material for: Factor quinolinone inhibitors alter cell morphology and motility by destabilizing interphase microtubules
Source: Sci Rep. 2021 Dec 7;11:23564. doi: 10.1038/s41598-021-02962-0 (PMC8651680; doi:10.1038/s41598-021-02962-0)
Supplement: Supplementary file 7 — Supplementary Information. [file 41598_2021_2962_MOESM7_ESM.pdf]

SUPPLEMENTARY INFORMATION

Factor Quinolinone Inhibitors Alter Cell Morphology and Motility by Destabilizing Interphase Microtubules

Patrick Stoiber, Pietro Scribani Rossi, Niranjana Pokharel, Jean-Luc Germany, Emily A. York, Scott E. Schaus, Ulla Hansen

Table of Contents

SUPPLEMENTARY METHODS ... 2

FQI2-34 Synthesis and Characterization ... 2

MTS Cell Proliferation Assay ... 5

Cellular Thermal Shift Assay (CETSA) ... 6

SUPPLEMENTARY FIGURES ... 7

Figure S1. FQI1-induced decrease in stable microtubule levels is reversible and not prevented by ROCK inhibitor ... 7

Figure S2. FQI1-induced cell compaction in FH-B cells is reversible and not due to mitotic arrest, with taxol preventing both microtubule destabilization and MLC2 phosphorylation. ... 8

Figure S4. Visible microtubule ends in FH-B cells are similarly increased by FQI1, whether proximal or distal to the centrosome in the cell. ... 10

Figure S5. Effects of FQI1 on RPE cells in wound healing and cell motility assays. ... 11

Figure S6. FQI2-34 compacts FH-B cells and inhibits RPE cell migration at a 20-fold lower concentration relative to that of FQI1. ... 12

Figure S7. Full immunoblots of data shown in Figures 1a, 1c, 1e, S1a, S1b, S1c, S1d, S2c, S2d, and S5g ... 16

Figure S8. Synthesis of FQI2-34. ... 17

SUPPLEMENTARY MOVIES (see separate files) ... 18

Movies 1 & 2: FQI1-treated FH-B cells undergo rapid morphological compaction. ... 18

Movies 3-6: FQI1 inhibits motility of FH-B and RPE cells. ... 18

SUPPLEMENTARY METHODS

FQI2-34 Synthesis and Characterization (see also Supplementary Fig. S8)

^{1}H NMR spectra were obtained at 400 MHz and referenced to the $CHCl_3$ singlet at 7.26 ppm, or the DMSO singlet at 2.50 ppm. ^{13}C NMR spectra were obtained at 100 MHz, and referenced to the center peak of the $CDCl_3$ triplet at 77.16 ppm, or the center of the DMSO-d_6 septet at 39.51 ppm. Chemical shifts are reported in parts per million as follows: chemical shift, multiplicity (s = singlet, d = doublet, t = triplet, q = quartet, m = multiplet), coupling constant, and integration. High resolution mass spectrometry data were obtained on a Waters Qtof (hybrid quadrupolar/time-of-flight) API US system by electrospray (ESI) in the positive mode. Mass correction was done by an external reference using a Waters Lockspray accessory. Mobile phases were water and acetonitrile with 0.1% formic acid. The MS settings were: capillary voltage = 3kV, cone voltage = 35, source temperature = 120 °C and dissolvation temperature = 350 °C. Flash column chromatography was performed on Sorbent Technologies 60 Å silica gel.

FQI-34 and FQI2-34 were generally prepared according to patented procedures (Hansen et al., 2019; Schaus et al., 2020), with a few modifications as detailed in the following step-by-step protocol (Scheme S1).

N-(benzo[d][1,3]dioxol-5-yl)-2-bromoacetamide: A flame-dried 500-mL round bottomed flask equipped with a Teflon-coated magnetic stirbar under an argon atmosphere was charged with 3,4-(methylenedioxy)aniline (5.88 g, 42.9 mmol) and dry dichloromethane (100 mL, 0.43 M). (Note: 3,4-(methylenedioxy)aniline was recrystallized from hexanes prior to use). Vacuum oven-dried potassium carbonate (8.30 g, 60 mmol, 1.40 equiv) was added, and the reaction was cooled to 0 ºC. Bromoacetyl chloride (4.64 mL, 55.7 mmol, 1.30 equiv) was added *via* syringe and the reaction was stirred at 0 ºC for 20 min and then allowed to warm to room temperature. Saturated aqueous sodium bicarbonate was added (100 mL) and the mixture was extracted with dichloromethane (3 x 150 mL). The combined organic layers were washed with saturated aqueous sodium chloride (100 mL), and dried over anhydrous sodium sulfate (Na_2SO_4). The filtrate was concentrated *via* rotary evaporation to afford N-(benzo[d][1,3]dioxol-5-yl)-2-bromoacetamide as a tan solid (9.73 g, 37.7 mmol, 88% yield, >97% purity), that was used without purification. ^{1}H NMR (DMSO-d_6, 400 MHz) δ 10.30 (s, 1H), 7.27 (d, J = 2.0 Hz, 1H), 6.96 (dd, J = 8.4, 2.0 Hz, 1H), 6.87 (d, J = 8.4 Hz, 1H), 5.98 (s, 2H), 4.00 (s, 2H). ^{13}C NMR (DMSO-d_6, 100 MHz) δ 164.4,

147.1, 142.9, 133.0, 112.2, 108.1, 101.3, 101.1, 30.4. HRMS *m/z* 257.9771 [(M + H^+) calculated for $C_9H_9BrNO_3^+$: 257.9766].

Dimethyl (2-(benzo[d][1,3]dioxol-5-ylamino)-2-oxoethyl)phosphonate: A flame dried 50-mL round-bottomed flask equipped with a Teflon-coated magnetic stirbar under an argon atmosphere was charged with N-(benzo[d][1,3]dioxol-5-yl)-2-bromoacetamide (4.00 g, 15.5 mmol) and trimethyl phosphite (9.62 g, 9.16 mL, 77.5 mmol). The flask was fitted with a reflux condenser, rubber septum, and argon balloon. The reaction was heated to 100 °C for 2 hours, at which time the reaction was poured into a 500-mL separatory funnel, diluted with dichloromethane (250 mL), and washed with water (3 x 200 mL). The organic layer was rinsed with saturated aqueous sodium chloride (50 mL), and dried over anhydrous sodium sulfate (Na_2SO_4). The filtrate was concentrated in vacuo to afford a viscous oil that was left under high vacuum (0.4 mmHg) overnight. Then, the product was dissolved in dry toluene (50 mL) and concentrated *via* rotary evaporation to azeotrope any remaining trimethyl phosphite. The product was further dried under high vacuum (0.4 mmHg) to afford dimethyl (2-(benzo[d][1,3]dioxol-5-ylamino)-2-oxoethyl)phosphonate as a pale pink/purple solid (3.43 g, 77% yield, >98% purity) that was used without further purification. ^{1}H NMR ($CDCl_3$, 400 MHz) δ 8.89 (s, 1H), 7.18 (d, J = 2.1 Hz, 1H), 6.79 (dd, J = 8.4, 2.1 Hz, 1H), 6.65 (d, J = 8.4 Hz, 1H), 5.91 (s, 2H), 3.82 (d = 11.2 Hz, 6H), 3.01 (d, J = 21.0 Hz, 2H). ^{13}C NMR ($CDCl_3$, 100 MHz) δ 161.7, 147.5, 144.0, 132.3, 112.8, 107.8, 102.4, 53.4, 35.9, 34.6. HRMS *m/z* 288.0630 [(M + H^+) calculated for $C_{11}H_{15}BrNO_6P^+$: 288.0637].

4-(dimethylamino)-2-ethoxybenzaldehyde: A flame-dried 250-mL round-bottomed flask equipped with a Teflon-coated magnetic stirbar under an argon atmosphere was charged with 4-(dimethylamino)-2-hydroxy-benzaldehye (4.13 g, 25 mmol) in acetone (83 mL, 0.30 M). Anhydrous potassium carbonate (5.18 g, 37.5 mmol), 18-crown-6 (330 mg, 1.25 mmol) and bromoethane (13.6 g, 125 mmol, 9.27 mL) were added to the reaction. The reaction mixture was fitted with a reflux glycol condenser and heated at 40 ºC for 16 hours, then cooled to room temperature. The reaction was filtered and the filtered solid was washed with acetone. The filtrate was evaporated *via* rotary evaporation to yield 4-(dimethylamino)-2-ethoxy-benzaldehyde as a brown solid (3.87g, >99% yield, 90% pure) that was used without further purification. ^{1}H NMR ($CDCl_3$, 400 MHz) δ 10.20 (s, 1H), 7.72 (d, J = 8.8 Hz, 1H), 6.29 (dd, J = 8.8, 2.3 Hz, 1H), 6.02 (d, J = 2.3 Hz, 1H), 4.11 (q, J = 7.0 Hz, 2H), 3.06 (s, 6H), 1.46 (t, J = 7.0 Hz, 3H). ^{13}C NMR

($CDCl_3$, 100 MHz) 187.6, 163.4, 155.9, 129.8, 114.6, 104.50, 93.7, 70.0, 63.7, 40.2, 14.7. HRMS *m/z* 194.1176 [(M + H^+) calculated for $C_{11}H_{16}NO_2^+$: 194.1181].

(E)-N-(benzo[d][1,3]dioxol-5-yl)-3-(4-(dimethylamino)-2-ethoxyphenyl)acrylamide: A flame dried 25-mL round-bottomed flask equipped with a Teflon-coated magnetic stirbar was charged with dimethyl (2-(benzo[d][1,3]dioxol-5-ylamino)-2-oxoethyl)phosphonate (718 mg, 2.50 mmol) and THF (8.3 mL, 0.30 M). The flask was flushed with argon and fitted with a rubber septum and argon balloon, then cooled to 0 °C in an ice-water bath. n-Butyllithium (1.6 M in hexanes, 1.35 equiv) was added dropwise, and the mixture was allowed to warm to RT and stir for 30 min, at which time 4-(dimethylamino)-2-ethoxybenzaldehyde (387 mg, 2.0 mmol) was added as a single portion. The flask was fitted with a reflux condenser and argon balloon, and the mixture was heated to reflux for 22 h. The mixture was cooled to RT and quenched with saturated ammonium chloride (5 mL). The mixture was transferred to a 500-mL separatory funnel, and diluted with dichloromethane (150 mL) and water (50 mL). The dichloromethane layer was removed and washed with water (3 x 50 mL), then dried over anhydrous sodium sulfate (Na_2SO_4), filtered, and concentrated by rotary evaporation. The product was isolated as a yellow brown solid (671 mg, 95% yield, 5:1 E:Z) and used without further purification. ^1H NMR ($CDCl_3$, 400 MHz) δ 7.90 (d, J = 15.3 Hz, 1H), 7.07 (s, 1H), 7.35 (d, J = 8.2 Hz, 1H), 6.84 (d, J = 8.2 Hz, 1H), 6.73 (d, J = 8.5 Hz, 1H), 6.44 (d, J = 15.3 Hz, 1H), 6.27 (dd, J = 8.5, 2.4 Hz, 1H), 6.14 (d, J = 2.4 Hz, 1H), 5.93 (s, 2H), 4.09 (q, J = 6.9 Hz, 2H), 3.00 (s, 6H), 1.47 (t, J = 6.9 Hz, 3H). ^{13}C NMR ($CDCl_3$, 100 MHz) 165.9, 159.7, 152.7, 147.6, 138.0, 135.2, 133.2, 130.3, 115.8, 112.8, 112.2, 108.0, 104.6, 102.7, 101.1, 97.2, 95.5, 63.7, 40.3, 14.9. HRMS *m/z* 355.1650 [(M + H^+) calculated for $C_{20}H_{23}N_2O_4^+$: 355.1650].

FQI-34 (8-(4-(dimethylamino)-2-ethoxyphenyl)-7,8-dihydro-[1,3]dioxolo[4,5-g] quinolin-6(5H)-one): A flame-dried 25-mL round-bottomed flask equipped with a Teflon-coated magnetic stirbar was charged with (E)-N-(benzo[d][1,3]dioxol-5-yl)-3-(4-(dimethylamino)-2-ethoxyphenyl)acrylamide (580 mg, 1.64 mmol) and trifluoroacetic acid (11 mL, 0.15 M). The flask was flushed with argon, and fitted with a reflux condenser, a rubber septum, and an argon balloon and refluxed for 20 h. The resulting mixture was cooled to room temperature, and transferred to a 500-mL Erlenmeyer flask. The mixture was diluted with dichloromethane (150 mL) and cooled to 0 ºC in an ice bath. The reaction mixture was quenched with saturated aqueous sodium bicarbonate (~100 mL) and then transferred to a 500-mL separatory funnel. The organic layer was removed,

and the aqueous layer was extracted with dichloromethane (2 x 50 mL). The combined organic layers were washed with saturated aqueous sodium chloride (50 mL), dried over anhydrous sodium sulfate (Na_2SO_4), filtered, and concentrated *via* rotary evaporation. The crude product was purified *via* column chromatography (gradient from hexanes to 1:1 hexanes:ethyl acetate) to afford the desired product as a pale orange-yellow solid (400 mg, 69% yield, >99% pure). ^{1}H NMR ($CDCl_3$, 400 MHz) δ 7.79 (s, 1H), 6.73 (d, J = 8.4 Hz, 1H), 6.47 (s, 1H), 6.35 (s, 1H), 6.27 (d, J = 2.4 Hz, 1H), 6.22 (J = 8.4, 2.4 Hz, 1H), 5.88 (s, 2H), 4.49 (dd, J = 7.0, 7.0 Hz, 1H), 4.05 (m, 2H), 2.94-2.87 (overlap, 7H), 2.76 (dd, J = 16.2, 6.4 Hz, 1H), 1.38 (t, 6.9 Hz, 3H). ^{13}C NMR ($CDCl_3$, 100 MHz) δ 171.8, 157.1, 151.0, 147.2, 143.9, 131.5, 128.6, 119.8, 117.8, 108.4, 104.7, 108.4, 104.7, 101.1, 97.6, 97.0, 63.4, 40.8, 37.0, 35.1, 14.9. HRMS *m/z* 355.1648 [(M + H^+) calculated for $C_{20}H_{23}N_2O_4^+$: 355.1658].

FQI2-34 (8-(4-(dimethylamino)-2-ethoxyphenyl)-[1,3]dioxolo[4,5-g]quinolin-6(5H)-one): A flame-dried 25-mL round-bottomed flask equipped with a Teflon-coated magnetic stirbar was charged with 8-(4-(dimethylamino)-2-ethoxyphenyl)-[1,3]dioxolo[4,5-g]quinolin-6(5H)-one (280 mg, 0.79 mmol) and 1,4-dioxane (0.06 M). DDQ (179 mg, 1.0 equiv) was added and the reaction was stirred at room temperature for 3 hours and then concentrated *via* rotary evaporation. The resulting residue was dissolved in 2.5% aqueous potassium carbonate solution (30 mL) and extracted with dichloromethane (3 x 50 mL). The combined organic layers were washed with saturated sodium chloride, (50 mL), dried over anhydrous sodium sulfate (Na_2SO_4), and concentrated. The solid crude product was purified *via* column chromatography (gradient from 80% ethyl acetate in hexanes to 100% ethyl acetate to afford the desired product as an off-white solid (203 mg, 73% yield, >99% pure). ^{1}H NMR ($CDCl_3$, 400 MHz) δ 12.14 (s, 1H), 7.10 (J = 8.4 Hz, 1H), 6.92 (s, 1H), 6.76 (s, 1H), 6.53 (s, 1H), 6.41 (dd, J = 8.4, 2.2 Hz, 1H), 6.33 (d, J = 2.2 Hz, 1H), 5.99 (m, 1H), 5.96 (m, 1H), 4.00 (q, J = 7.1 Hz, 2H), 3.04 (s, 6H), 1.19 (t, J = 7.1 Hz, 3H). ^{13}C NMR ($CDCl_3$, 100 MHz) δ 164.4, 156.8, 152.2, 151.5, 150.2, 143.9, 135.4, 131.2, 118.7, 115.4, 115.1, 105.1, 104.7, 101.5, 97.0, 96.2, 63.9, 40.5, 14.7. HRMS *m/z* 353.1494 [(M + H^+) calculated for $C_{20}H_{21}N_2O_4^+$: 353.1501].

MTS Cell Proliferation Assay

Three thousand FH-B cells were seeded per well in a 96-well plate and incubated at 37 °C for 20 hours. Compound dilutions were prepared in anhydrous DMSO and added to the cell culture media

to a final DMSO concentration of 1% for a 72 hour treatment. Cell growth inhibition was assessed by measuring absorbance at 490 nm using the Promega CellTiter 96 AQueous One Solution Cell Proliferation Assay (Promega, G358C). Percent growth inhibition was determined by the ratio of the absorbance of treatment wells to the absorbance of control wells. A non-linear regression plot of the percentage of growth inhibition versus compound concentration was prepared using GraphPad Prism software, using settings for the curves of variable slope and four parameters. The relative GI50 value was determined from the curve as the concentration of drug that provokes a response halfway between the top and bottom plateaus of the curve.

Cellular Thermal Shift Assay (CETSA)

Huh7 cells obtained from JCRB (Japanese Cancer Resources Bank) were cultured in DMEM medium (Corning) supplemented with 10% fetal bovine serum (Gibco). The cells were cultured at 37 °C in 5% CO_2. Approximately $9x10^5$ Huh7 cells were plated per 10 cm plate. After 20 hours, cells were treated for three hours with fresh media containing 50 μM FQI1, 50 μM FQI2-34, or 0.1% DMSO (FQI1 control) or 0.5% DMSO (FQI2-34 control). Subsequently, the cells were washed with PBS (137 mM NaCl, 2.7 mM KCl, 10 mM Na_2HPO_4, 2 mM KH_2PO_4, pH 7.2) supplemented with their respective treatments (FQI1, FQI2-34, or vehicle) and scraped from the dish in the presence of 5 mL of PBS supplemented with 1 mM Pefabloc (Sigma-Aldrich, 76307) plus the respective treatment. Pelleted cells were resuspended in 500 μL of PBS buffer with 1 mM Pefabloc plus the respective treatment. Aliquots of 50 μL of cell suspension from each treatment sample were incubated separately at the indicated temperatures for 3 minutes in a thermal cycler (Bio-rad, T100 Thermocyler), then cooled at the room temperature for 3 minutes. Lysates were prepared by four rounds of snap freezing and thawing. Soluble protein was separated from aggregates by centrifugation at 20,000 x g for 10 minutes. 40 μL of each supernatant was used for immunoblot analysis. Lysates were separated by electrophoresis through 10% SDS polyacrylamide gels, in 25 mM Tris, 192 mM glycine, and 0.1% SDS. Gels were transferred to PVDF membranes and incubated in blocking buffer with 5% milk in TBST for 1 hour. Membranes were incubated overnight with anti-LSF antibody (1:1,000, BD Bioscience, 610818) at 4°C, and subsequently with goat anti-mouse HRP antibody (Thermo Fisher Scientific, 62-6520; 1:7,000) for 1 hour at room temperature. Films were scanned and band intensities were quantified by densitometry with Image J; a non-parametric t-test was used to determine statistical significance.

SUPPLEMENTARY FIGURES

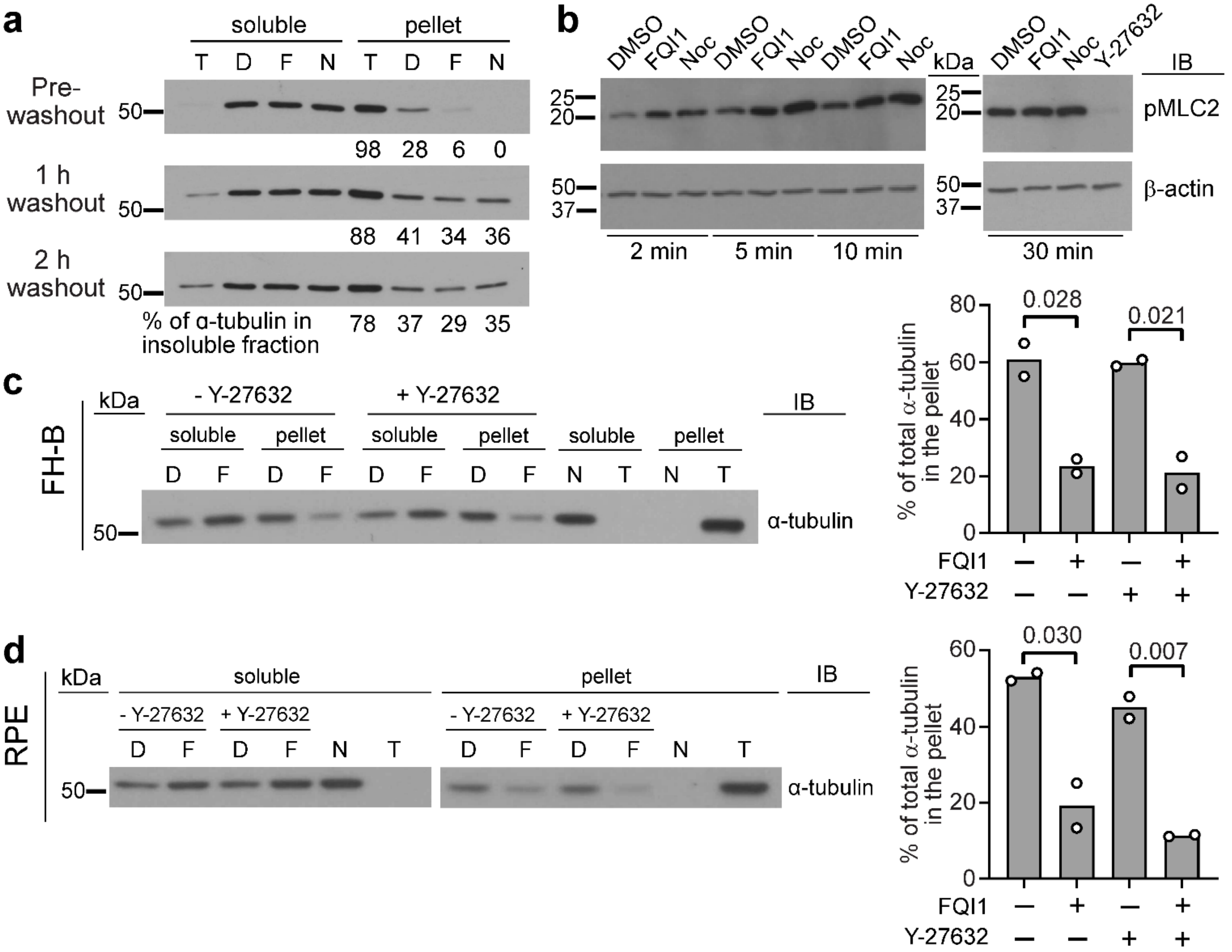

Figure S1. FQI1-induced decrease in stable microtubule levels is reversible and not prevented by ROCK inhibitor.

(**a**) FH-B cells were treated with fresh media containing indicated inhibitors (1 μM Taxol, 4 μM FQI1, 1 μM nocodazole) or vehicle (0.01% DMSO) for 1 hour. Cells were either harvested immediately (pre washout) or incubated with fresh media for 1 hour or 2 hours, followed by the microtubule sedimentation assay. Quantitation of the levels of pelleted tubulin relative to total tubulin are shown underneath each immunoblot. The experiment was performed once. (**b**) FH-B cells were treated with 4 μM FQI1, 1 μM nocodazole, 10 μM Y-27632, or vehicle (0.01% DMSO) for 2, 5, 10, or 30 minutes. Cell lysates were blotted for pMLC2 and β-actin. (**c,d**) FH-B and RPE cells were pretreated with 10 μM Y-27632 or vehicle (0.01% DMSO) for 30 minutes, followed by treatment with either 4 μM FQI1 or vehicle (0.02% DMSO) for another 30 minutes. As positive and negative controls, taxol ("T", 1 μM) and nocodazole ("N", 1 μM), respectively, were also added after the pretreatment. Cells were then fractionated using the microtubule sedimentation assay, followed by an analysis of soluble and insoluble α-tubulin. Two independent biological replicates were performed for each cell line. Left panels: Representative α-tubulin immunoblots of FH-B (**c**) and RPE (**d**) lysate fractions from the DMS0- and FQI1-treated cells. Right panels: quantitation of immunoblots. Numbers above brackets represent p-values, which were calculated using an unpaired two-sample t-test. Bars represent means and circles represent individual data points. For full immunoblots of (**a**), (**b**), (**c**), and (**d**), see Supplementary Fig. S7.

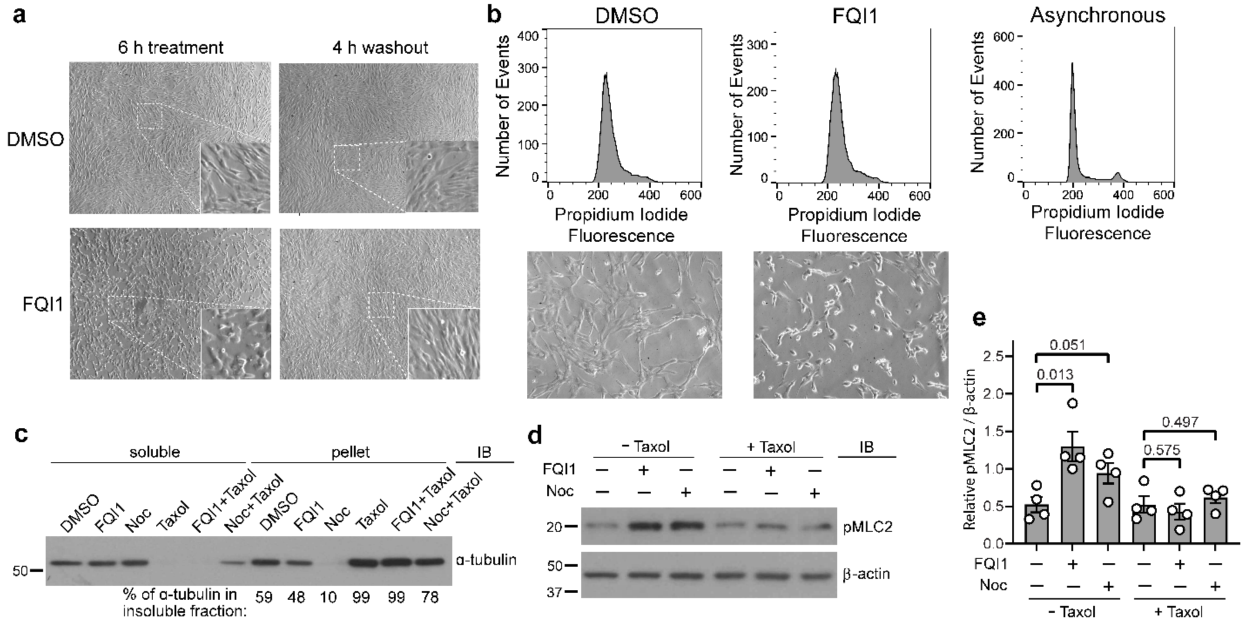

Figure S2. FQI1-induced cell compaction in FH-B cells is reversible and not due to mitotic arrest, with taxol preventing both microtubule destabilization and MLC2 phosphorylation.
(**a**) FH-B cells were pretreated with 2 mM thymidine for 18 hours, then for 6 hours with fresh media containing 2 mM thymidine plus either 3.6 μM FQI1 or vehicle (1% DMSO). Finally, media was replaced for a 4-hour treatment with fresh media. Phase contrast images were taken after 6 hours of treatment and after 4 hours of washout. Inserts depict representative areas of the image, magnified by 150%. The experiment was performed once. (**b**) Representative flow cytometry analysis of cellular DNA content showing cell cycle profiles of thymidine-blocked FH-B cells treated with 4 μM FQI1 or vehicle (0.01% DMSO) for 1 hour (upper row), along with phase contrast images of cells taken prior to harvesting and fixation (lower row). FH-B cells in this experiment received fresh media along with the respective treatments. Phase contrast images were taken using a 10x objective on an Olympus IX50 microscope. Two biological replicates exhibited identical findings. (**c**) Immunoblot of α-tubulin in soluble and insoluble microtubule sedimentation assay fractions harvested from FH-B cells treated for 1 hour with fresh media containing either 0.01% DMSO, 4 μM FQI1 (0.01% DMSO), 1 μM nocodazole (0.01% DMSO), 1 μM taxol (0.01% DMSO), 4 μM FQI1 plus 1 μM taxol (0.02% DMSO), or 1 μM nocodazole plus 1 μM taxol (0.02% DMSO). Quantitation of the percentage of the total α-tubulin in the insoluble fractions is presented below the gel image. The experiment was performed once. (**d**,**e**) FH-B cells were pretreated with vehicle (0.01% DMSO) or 1 μM taxol for 30 minutes, and then treated with vehicle (0.02% DMSO), 4 μM FQI1 or 1 μM nocodazole for 10 minutes. (**d**) A representative immunoblot of cell lysates probed for phospho-MLC2 (threonine 18, serine 19) and β-actin. (**e**) Quantitation of levels of phospho-MLC2 intensity relative to β-actin intensity in all treatment groups from four independent experiments. Bars and error bars represent the mean ± s.e.m; circles represent individual data points. Numbers above brackets represent p-values, which were calculated using an unpaired two-sample t-test. For full immunoblots of (**c**) and (**d**), see Supplementary Fig. S7.

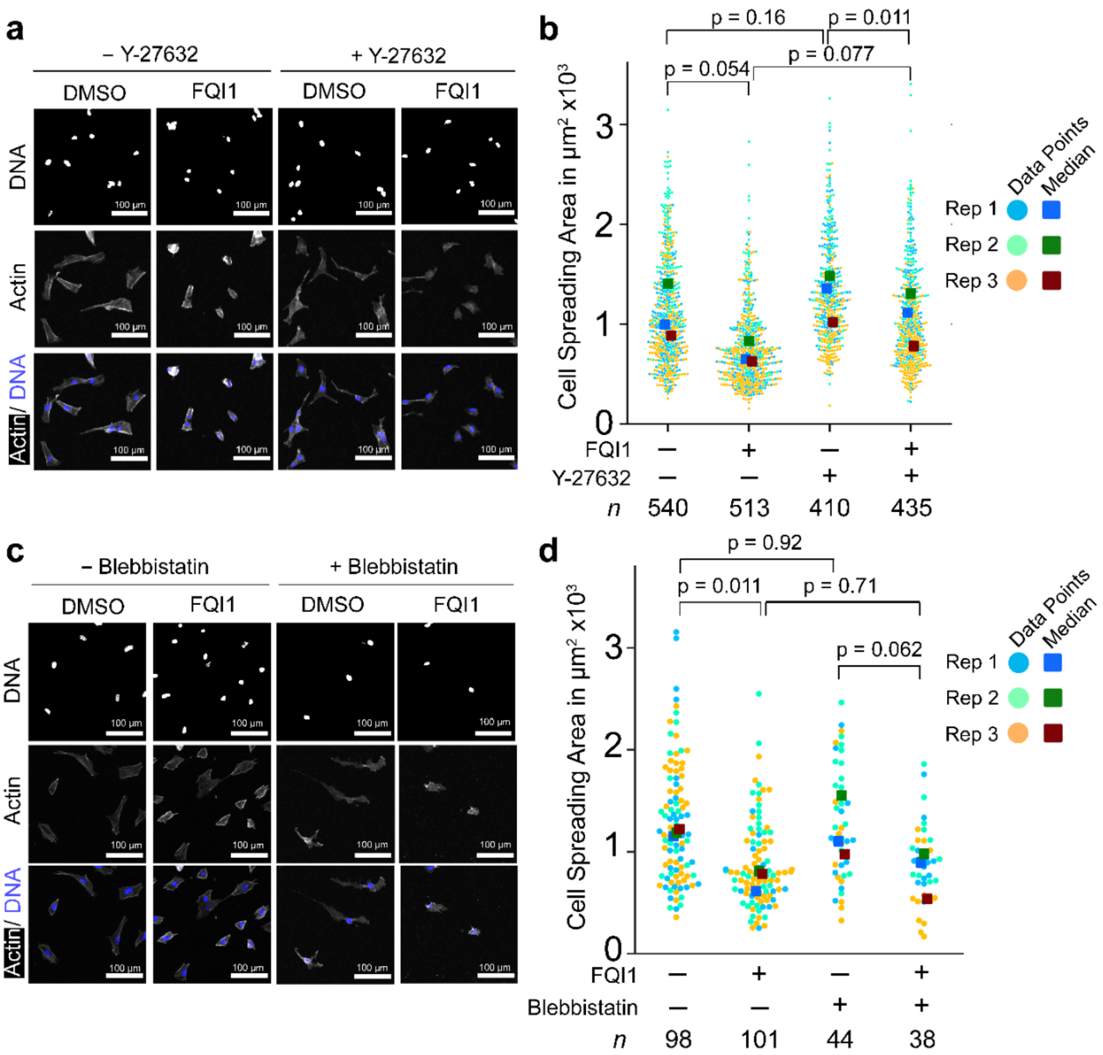

Figure S3. Activation of the myosin II pathway may be only partially responsible for FQI1-induced cell compaction.

(**a,b**) FH-B cells were pretreated with either Y-27632 or vehicle (0.01% DMSO) for 30 minutes, then were incubated in either 4 μM FQI1 or vehicle (0.02% DMSO) for another 30 minutes. (**a**) Representative fluorescence images of FH-B cells from the four treatment groups, which were fixed and stained with fluorescently-labelled Phalloidin and Hoechst 33342. Images were analyzed using ImageJ. (**b**) Swarmplots depict cell spreading area of individual cells and medians from each treatment group. Paired t-tests were performed between DMSO- and FQI1-treated cells from both pretreatment groups. Data were pooled from three independent biological replicates. The total number of cells analyzed in each condition is indicated by "*n*". When calculated in bulk using the Mann-Whitney U test, the p values for +/- FQI1 were < 2.2×10^{-16} in the absence of Y-27632, and 3.7×10^{-14} in the presence of Y-27632, and the p values for +/- Y-27632 were 7.1×10^{-9} in the absence of FQI1 and < 2.2×10^{-16} in the presence of FQI1. (**c,d**) FH-B cells were pretreated with fresh media containing either 50 μM Blebbistatin (Sigma Aldrich, B0560) or vehicle (0.05% DMSO) for 30 minutes, followed by a 1-hour treatment with either 4 μM FQI1 or vehicle (0.06% DMSO). Cells were then fixed, stained for actin and DNA, and imaged. (**c**) Representative fluorescence images of actin- and DNA-stained FH-B cells from all four treatment groups. (**d**) Swarmplots of cell spreading area of individually discernible cells from each treatment group across three independent experiments. P-values were calculated using a paired t test between medians from each replicate. When calculated in bulk using the Mann-Whitney U test, the p values for +/- FQI1 were 9.8×10^{-8} in the absence of blebbistatin, and 5.0×10^{-4} in the presence of blebbistatin, and the p values for +/- blebbistatin were 0.77 in the absence of FQI1 and 0.62 in the presence of FQI1. The number of cells analyzed in each condition is indicated by "n".

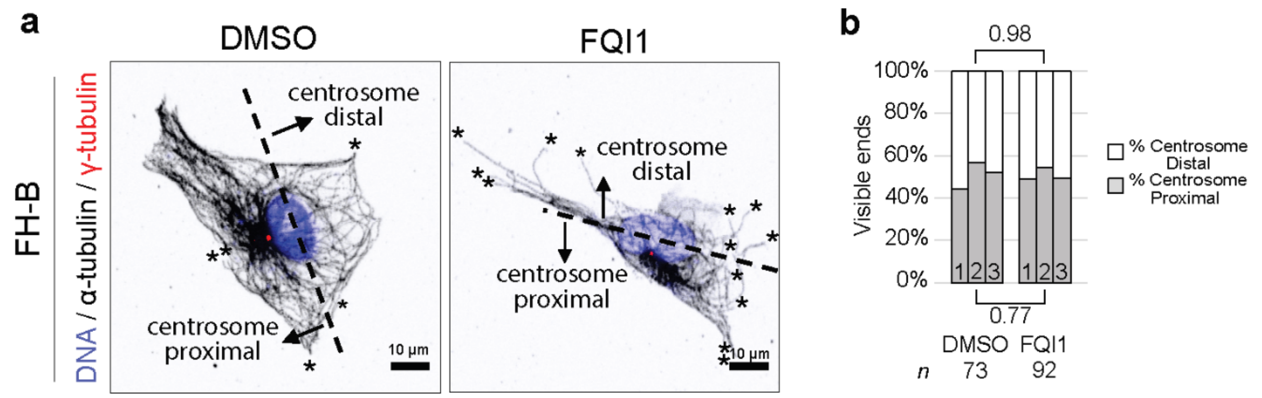

Figure S4. Visible microtubule ends in FH-B cells are similarly increased by FQI1, whether proximal or distal to the centrosome in the cell.

Representative fluorescence images of digitally magnified FH-B (**a**) cells are depicted. Dashed lines separating centrosome proximal and centrosome distal portions of the cells were generated using γ-tubulin and Hoechst localization as guides. Visible microtubule ends, marked with asterisks, were counted as in Fig. 2d,f, and partitioned into percentages in the two sectors, which were averaged and plotted as stacked bar charts (**b**). P-values for the centrosome distal and centrosome proximal percentages are indicated above and below the brackets, respectively and were determined using an unpaired two-sample t-test comparing the averages for each group; numbers in stacked bar plots represent the indicated biological replicates as designated in Fig. 2d.

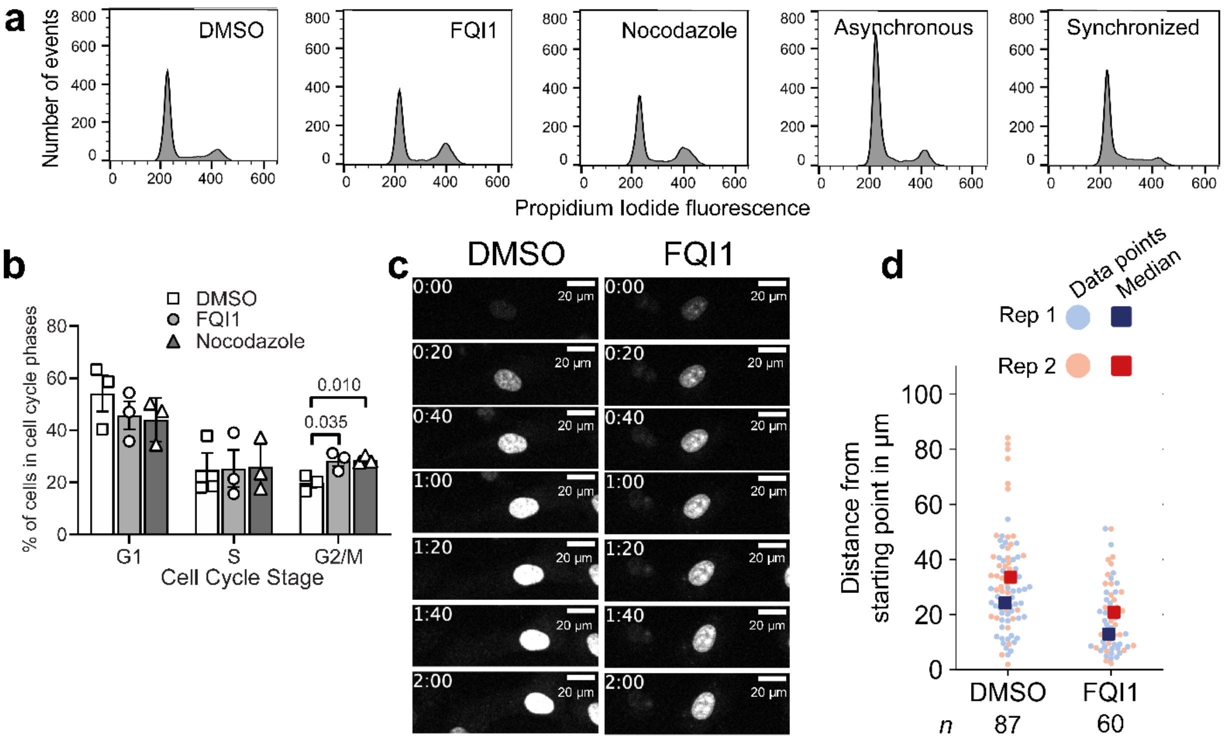

Figure S5. Effects of FQI1 on RPE cells in wound healing and cell motility assays.
(**a,b**) Confluent RPE cells treated with high concentrations of thymidine to limit proliferation both before and during the wound healing assay were analyzed for cellular DNA content at the final, 12-hour endpoint of the experiment (Fig. 4a,b). (**a**) Representative histograms of DNA content are shown across all treatment groups at 12 hours, plus controls that were harvested at 0 hours of an asynchronous population and a population of cells treated with 2 mM thymidine block. (**b**) Quantitation of the proportion of cells in G1, S, and G2/M cell cycle phases derived from the histograms presented in (**a**). Bar graphs and error bars represent the mean ± s.e.m. Three independent biological replicates were performed. Numbers above brackets represent p-values, which were calculated using an unpaired two-sample t-test. (**c,d**) RPE cells were synchronized, treated, and imaged the same way as FH-B cells in Fig. 5. Images of representative nuclei of migrating RPE cells from both treatment groups, taken at 20-minute intervals, are shown in (**c**) and the swarmplot of distances travelled by migrating RPE cells is shown in (**d**). Given only 2 biological replicates and the possibility of the apparent trend occurring by chance between the DMSO and FQI1-treated samples, a t-test was viewed as inappropriate. When calculated in batch using the Mann-Whitney U test, $p = 4.5\times10^{-6}$. The total number of cells analyzed in each condition is indicated by "*n*".

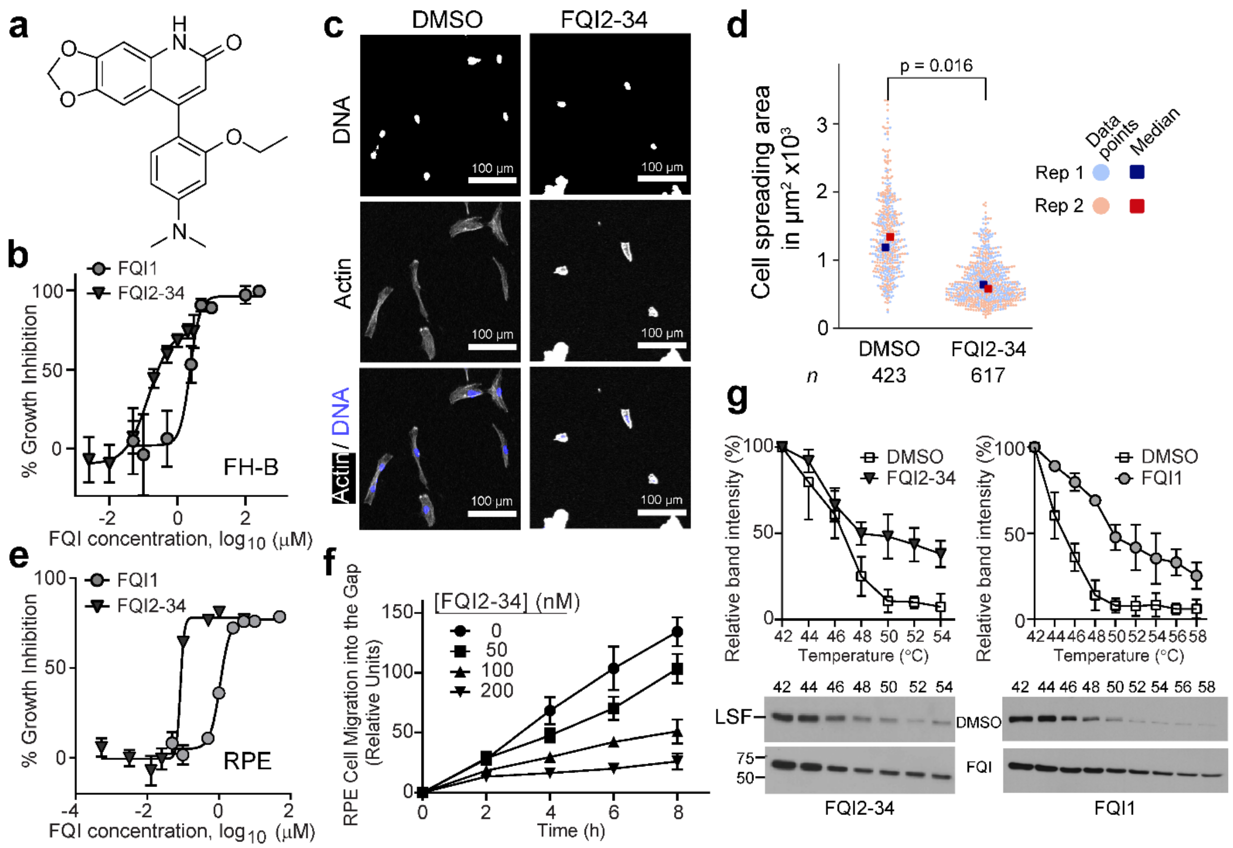

Figure S6. FQI2-34 compacts FH-B cells and inhibits RPE cell migration at a 20-fold lower concentration relative to that of FQI1.

(**a**) The structure of FQI2-34. (**b**) Comparison of sensitivities of FH-B cells to FQI1 versus FQI2-34, as determined by MTS assays. Shown are curves of cell viability over the indicated concentration ranges from six technical replicates. Data indicate means ± standard deviation. Calculated GI_{50} values are 2.3 μM for FQI1 and 0.14 μM for FQI2-34. (**c,d**) FH-B cells were treated with 200 nM FQI2-34 or vehicle (0.01% DMSO) for 30 minutes, fixed, stained with fluorescently labelled phalloidin and Hoechst 33342, and analyzed as in Fig. 1a,b. Representative fluorescence images are shown in (**c**). The areas covered by individual cells in each treatment group were quantified using ImageJ and displayed as swarmplots in (**d**). P-values were calculated using an unpaired two-sample t-test on medians from two biological replicates. When calculated in bulk using the Mann-Whitney U test, the p value was $< 2.2 \times 10^{-16}$ between DMSO and FQI1 treatments. The number of cells analyzed in each condition is indicated by "*n*". (**e**) Comparison of sensitivities of RPE cells to FQI1 versus FQI2-34, as determined by MTS assays. Shown are curves of cell viability over the indicated concentration ranges from six technical replicates. Data indicate means ± standard deviation. Calculated GI_{50} values are 1.3 μM for FQI1 and 79 nM for FQI2-34. (**f**) Cell migration of RPE cells in the wound healing assay was performed in the presence of 0-200 nM FQI2-34 (0.01% DMSO). Shown are a time course of measurements of the relative distances of cell migration following wounding. Means and standard deviations of technical triplicates are displayed. Data are representative of two independent biological experiments. Analysis with 2-tailed, paired t-tests indicated significant differences from the control as follows: 50 nM FQI2-34, p=0.004 (8h); 100 nM FQI2-34, p=0.042 (4h), 0.021 (6h), 0.021 (8h); 200 nM FQI2-34, p=0.037 (2h), 0.019 (4h), 0.015 (6h), 0.0059 (8h). (**g**) Cell extract thermal stability assays (CETSAs) were performed using Huh7 cells treated with either FQI1 or FQI2-34 as compared to treatment with vehicle alone (DMSO). Bottom panels: Representative immunoblots of soluble LSF after treatment with FQI2-34 versus DMSO (left) or FQI1 versus DMSO (right), after incubations at the indicated temperatures. Top panels: Quantitation of thermal stability of LSF after treatment of cells with each condition, as indicated. Data points are the mean ± standard deviations from a total of 3 independent experiments. For full immunoblots of (**g**), see Supplementary Fig. S7.

Fig. 1a

IB: α-tubulin

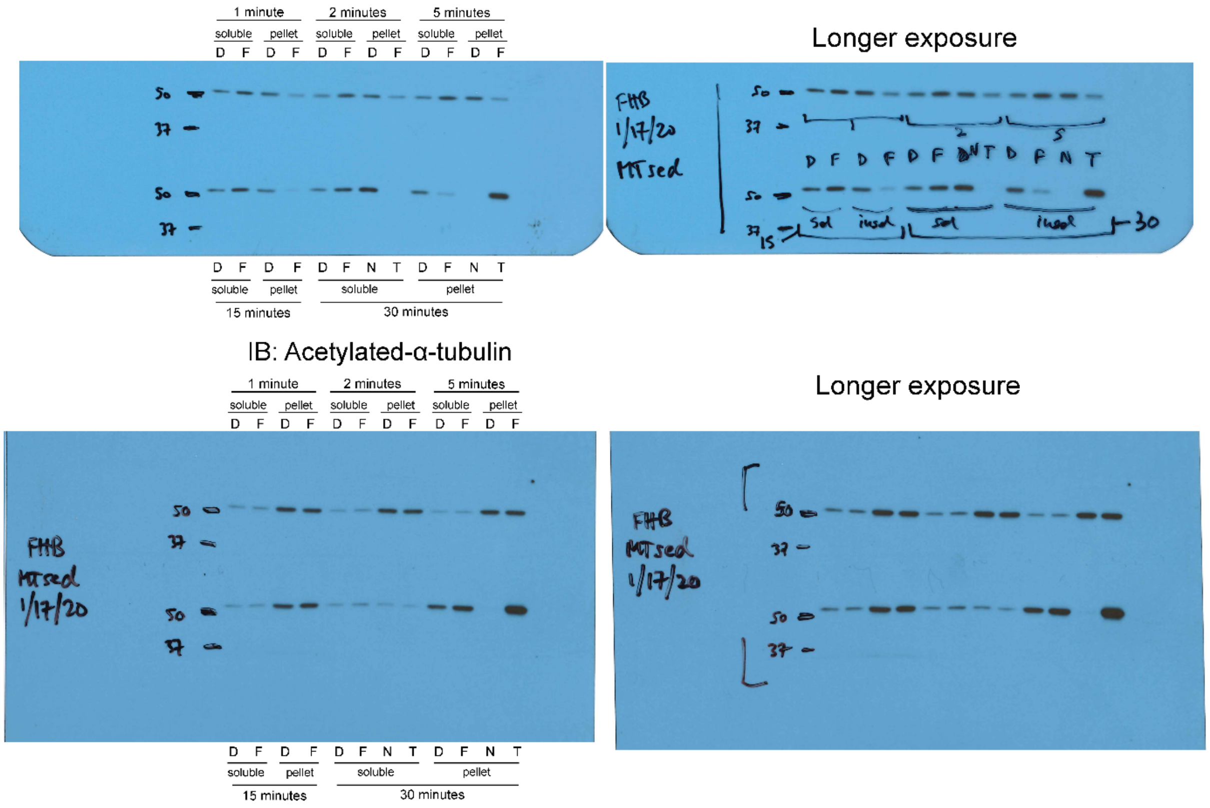

Fig. 1e

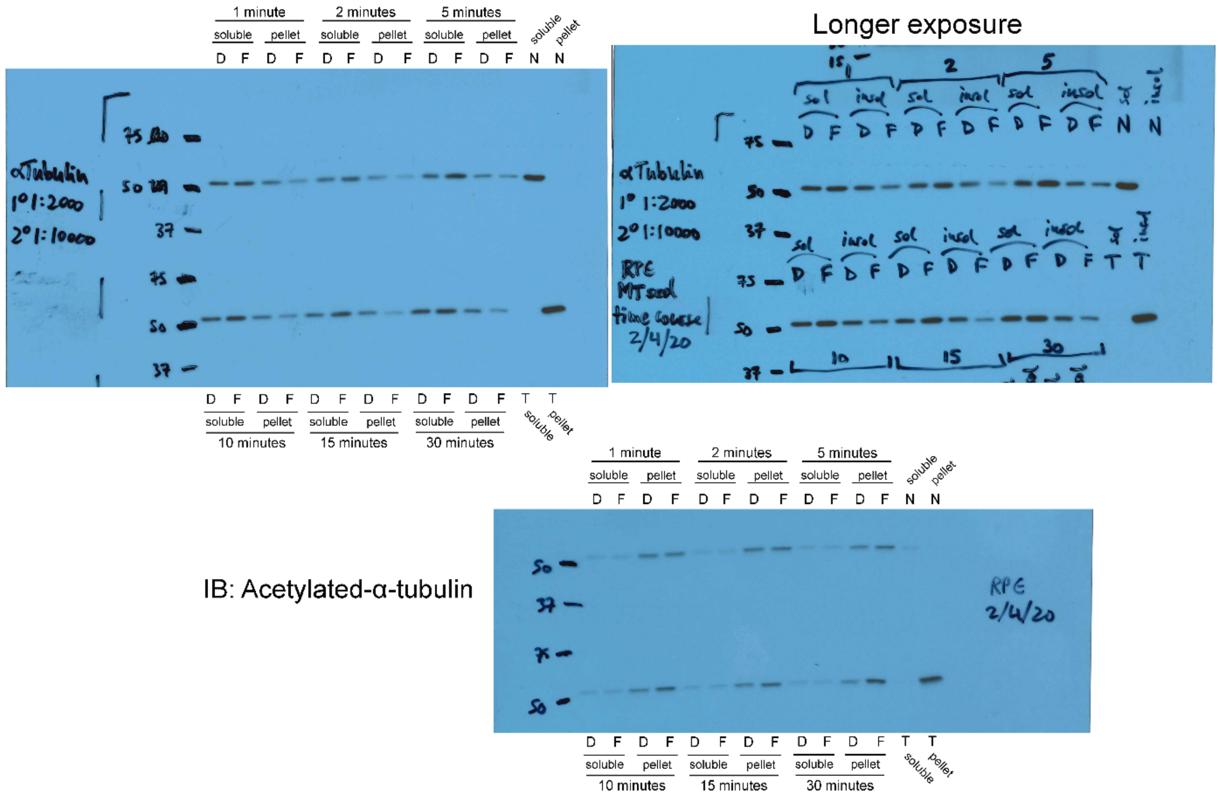

Fig. 1c

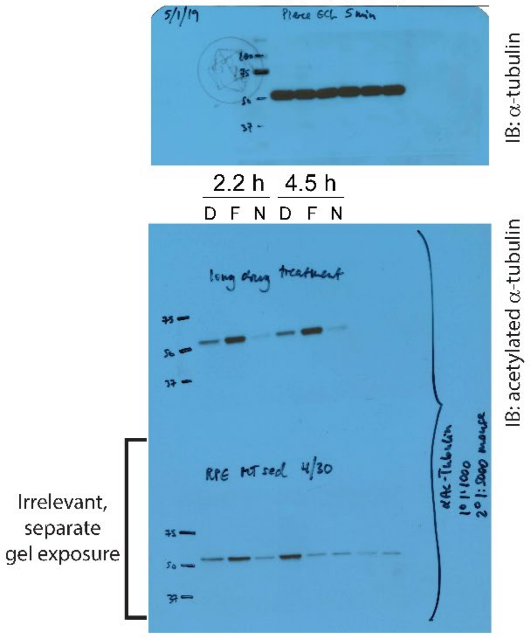

Fig. S1a

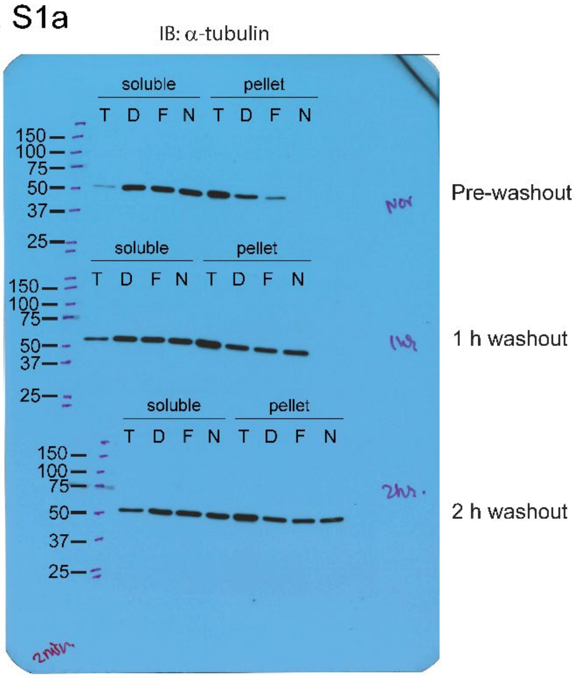

Fig. S1b

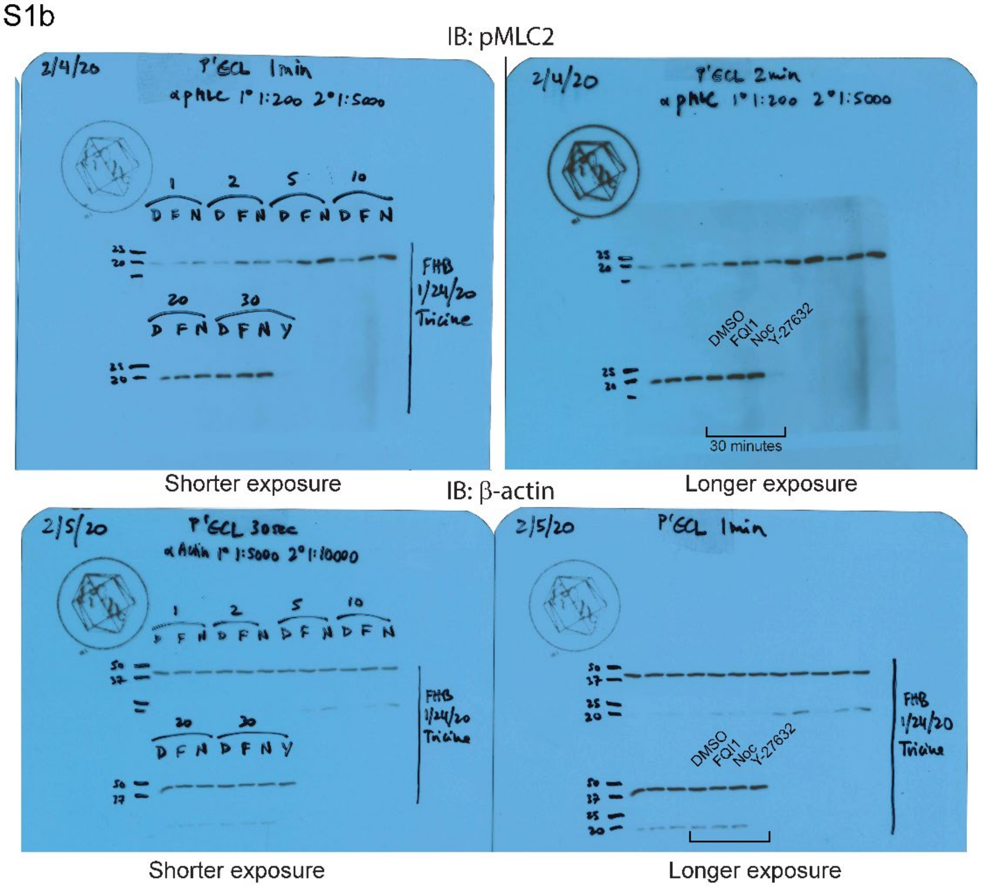

Fig. S1c

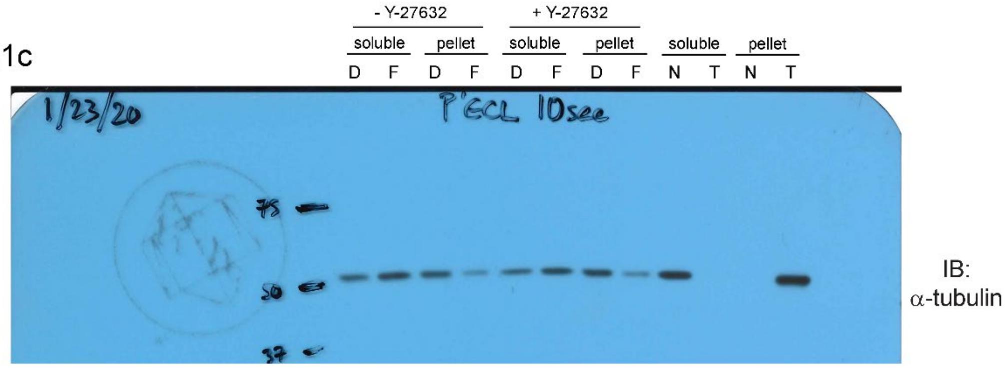

Fig. S1d

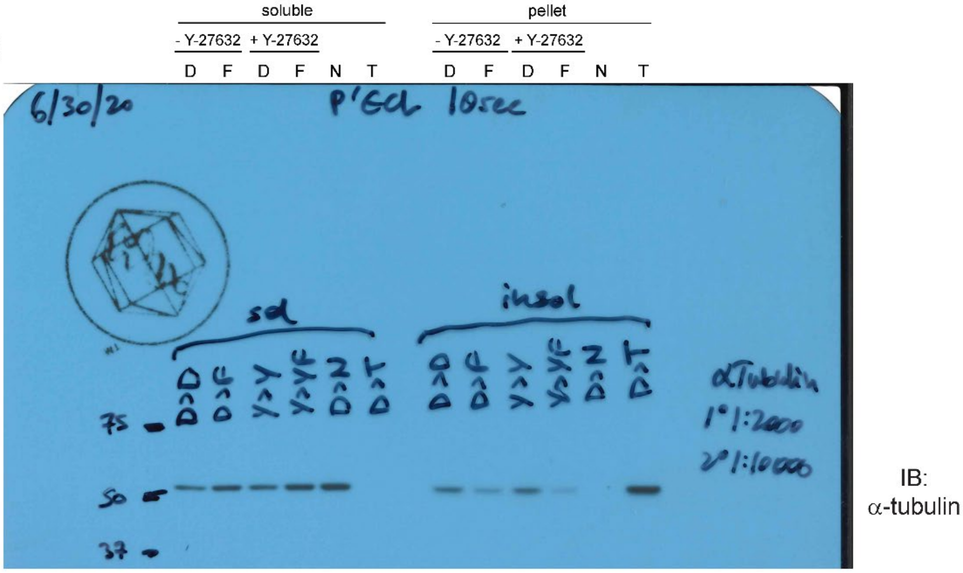

Fig. S2c

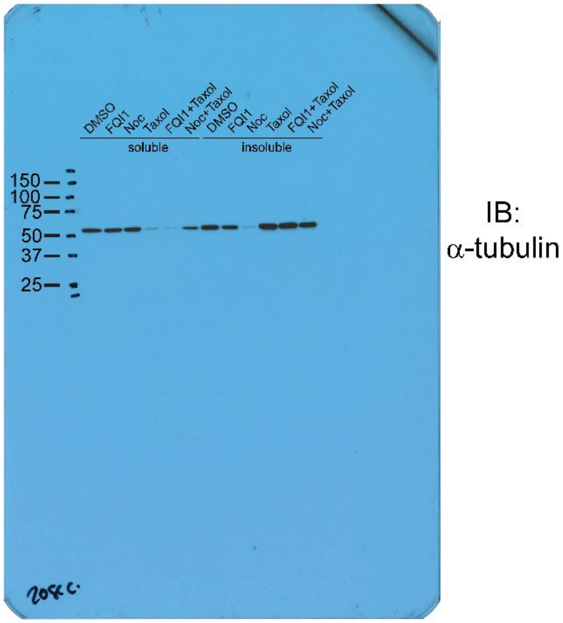

Fig. S2d

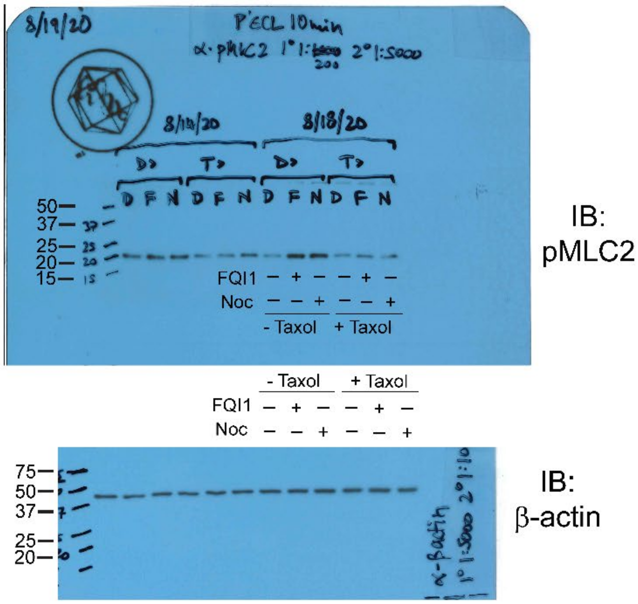

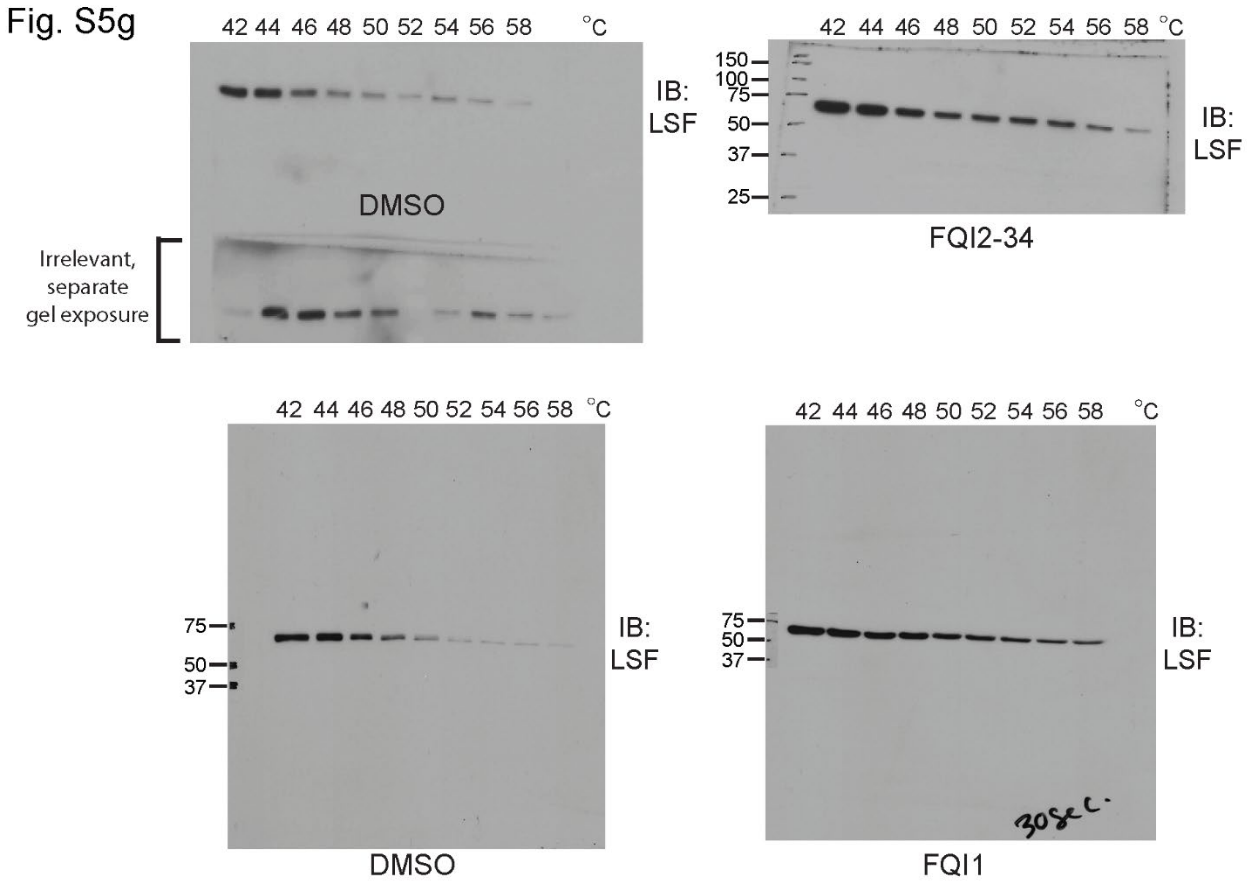

Figure S7. Full immunoblots of data shown in Figures 1a, 1c, 1e, S1a, S1b, S1c, S1d, S2c, S2d, and S5g.

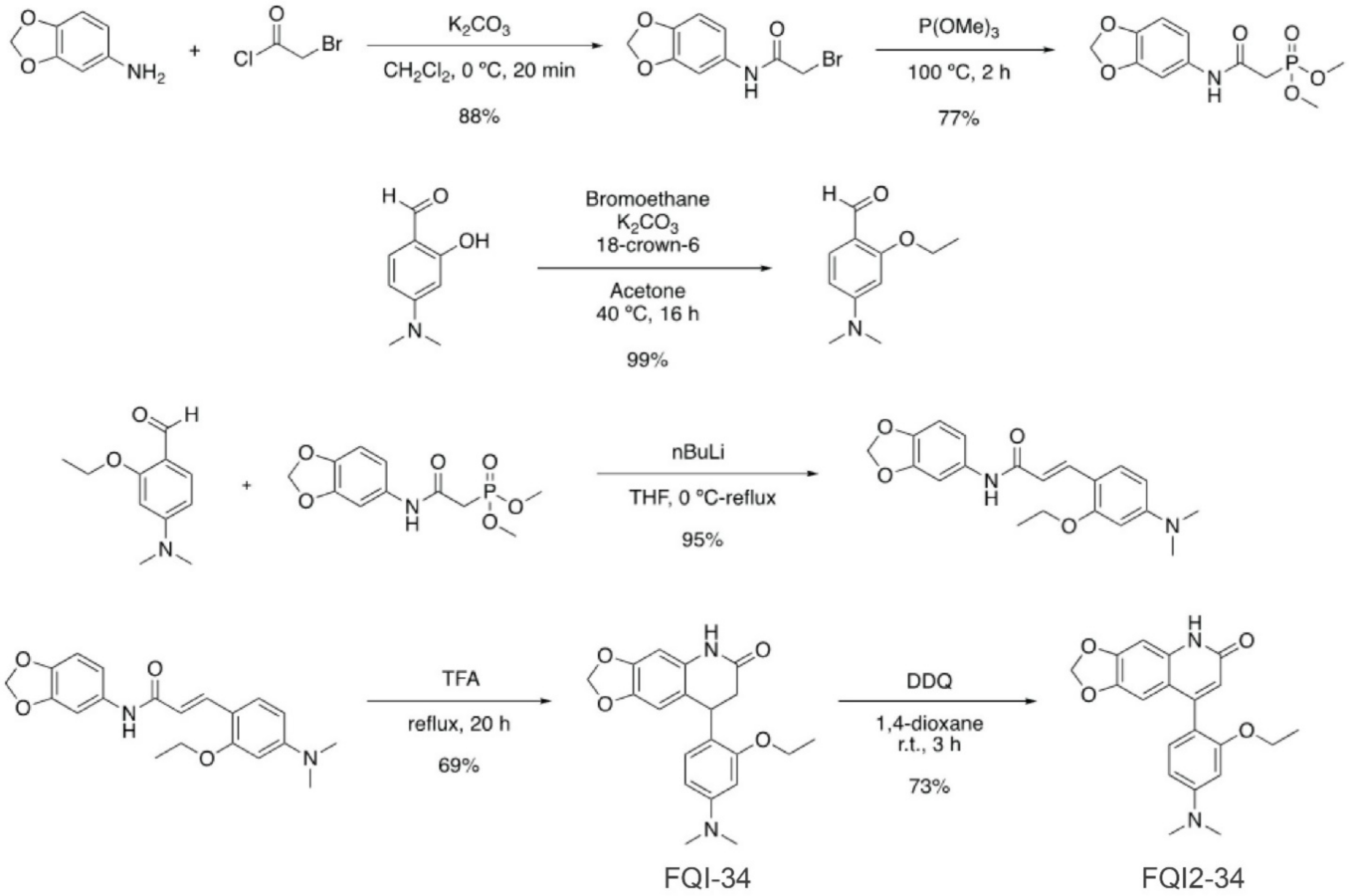

Figure S8. Synthesis of FQI2-34.
Indicated is a summary of the series of steps for synthesizing the final product, FQI2-34. Details are included in the Supplementary Methods (above). The yields for each step are indicated underneath each arrow.

SUPPLEMENTARY MOVIES (see separate files)

Movies 1 & 2: FQI1-treated FH-B cells undergo rapid morphological compaction.
FH-B cells were stained with CellBrite Steady Membrane and treated with either 0.01% DMSO (**Movie 1**) or 4 μM FQI1 (**Movie 2**). Cells were imaged by fluorescence time-lapse imaging every 30 seconds using a 20x objective. Asterisks denote cells that are presented in Fig. 3c. Frame rate was set to ten frames per second. Timestamps denote minutes:seconds. Scale bars are 50 μm.

Movies 3-6: FQI1 inhibits motility of FH-B and RPE cells.
FH-B and RPE cells were treated as described in Fig. 5c,d and Supplementary Fig. S5c,d, respectively. Briefly, cells were synchronized by a single thymidine block, treated with 4 μM FQI1 or vehicle (0.01% DMSO) for 1 hour, stained with NucSpot Live 650 dye and monitored by time-lapse fluorescence microscopy for 2 hours. Movies 3 and 4 show DMSO- and FQI1-treated FH-B cells, respectively; movies 5 and 6 show DMSO- and FQI1-treated RPE cells, respectively. Asterisks denote cells that are presented in Fig. 5c,d and Supplementary Fig. S5c,d. Frame rate was set to ten frames per second. Timestamps denote hours:minutes. Scale bars are 50 μm.
